# Crop yield responses to seaweed extract-based biostimulants depend on application strategy, formulation, and extraction methods: a meta-analysis

**DOI:** 10.3389/fpls.2026.1803269

**Published:** 2026-04-21

**Authors:** Christian José Pérez-Oñate, Mayara Cristina Malvas Nicolau, Tatiane Cristovam Ferreira, Fernando Broetto, João William Bossolani, Marco Montes de Oca, Willi Heitmann, Cleiton José Alves, Marcos de Oliveira Bettini, German Arturo Moreno-Poveda, Diego Villaseñor-Ortiz, Leonardo Cury da Silva, Samir Geraigire Filho, Holly Little, Sarah Maude, Marciel J. Stadnik

**Affiliations:** 1 Doctoral Program in Sustainable Agriculture, Universidad Nacional Agraria La Molina, Lima, Peru; 2 Department of Biology, Faculty of Agricultural and Veterinary Sciences, São Paulo State University (UNESP), Jaboticabal, Brazil; 3 Department of Rural Engineering, School of Agricultural Sciences, São Paulo State University (UNESP), Botucatu, Brazil; 4 Department of Chemistry and Biochemistry, Institute of Biosciences, São Paulo State University (UNESP), Botucatu, Brazil; 5 College of Agricultural Sciences (FCA), São Paulo State University (UNESP), Botucatu, Brazil; 6 Department of Bioinformatics, Neocrop Technologies SpA, Valdivia, Chile; 7 Master’s Program in Plant Sciences, Faculty of Agricultural Sciences, Universidad Austral de Chile, Valdivia, Chile; 8 Acadian Seaplants Limited, Dartmouth, NS, Canada; 9 Doctoral Program in Sciences, Universidad Nacional de Colombia, Bogotá, Colombia; 10 School of Agricultural Sciences, Universidad Técnica de Machala (UTMACH), Machala, Ecuador; 11 Postgraduate Program in Viticulture and Enology, Federal Institute of Rio Grande do Sul (IFRS), Bento Gonçalves, Brazil; 12 Department of Plant Sciences, School of Agricultural Sciences, Federal University of Santa Catarina (UFSC), Florianópolis, Brazil

**Keywords:** crop yield, extraction method, meta-analysis, seaweed extract, soil- environment interactions

## Abstract

Seaweed extract-based biostimulants have attracted sustained attention in agronomic research because of their biochemical diversity and their capacity to influence multiple plant processes. However, evidence on their yield effects remains fragmented across crops, environments, and management contexts. To address this gap, we synthesized data from 198 peer-reviewed studies encompassing 2,021 independent comparisons between seaweed extract treatments and untreated or conventionally managed controls. Crop yield and biomass responses were quantified using log response ratios and analyzed with inverse-variance weighted random-effects models estimated by restricted maximum likelihood. Variability among studies was explored in relation to climate, stress exposure, experimental setting, soil chemical properties, crop groups, phenological timing, application strategies, formulation characteristics, extraction methods, and algae species. Across all studies, seaweed extract applications increased yield by an average of 16.3% (95% CI: 13.5-19.1%), with positive responses observed under both stress and non-stress conditions. The largest effects occurred in tropical and arid regions and were most pronounced in vegetables, legumes, and cereals. Soil phosphorus and potassium availability, pH, salinity, and organic matter content influenced response magnitude but did not reverse the overall benefit. Higher and more consistent responses were associated with soil-directed applications, early-stage programs, powder formulations, and alkaline extraction processes. Among species, *Ascophyllum nodosum* showed the most stable effects, whereas responses linked to *Laminaria, Gracilaria*, and *Kappaphycus* were more variable. Overall, these findings indicate that seaweed extracts are associated with reproducible yield benefits while identifying agronomic conditions under which their effectiveness can be maximized.

## 1 Introduction

The increasing demand for more sustainable and resilient agricultural systems in the face of climate change and the depletion of natural resources has driven the development and adoption of biologically based inputs, including plant biostimulants, which have gained prominence (Du Jardin, 2015; Yakhin et al., 2017). Plant biostimulants are defined as substances or microorganisms applied to plants with the aim of stimulating natural processes that enhance nutrient uptake efficiency, tolerance to abiotic stress, and/or crop quality, regardless of the product’s nutrient content (Du Jardin, 2015). This widely accepted definition underscores the functional role of biostimulants, distinguishing them from fertilizers, chemical pesticides, and classical plant growth regulators, and supports their regulation as an autonomous category within the agricultural sector (Yakhin et al., 2017). Recent conceptual advances further emphasize that biostimulants are not defined by what they contain, but by what they do; they represent a transition from input-based to function-based agronomy, focusing on the enhancement of physiological processes rather than simple nutrient addition (Du Jardin et al., 2025).

Among the main categories of plant biostimulants, seaweed extracts stand out as the most extensively investigated and applied in agriculture due to their high biochemical complexity and recognized agronomic multifunctionality (Battacharyya et al., 2015; Shukla et al., 2019). These seaweed-derived products are rich in bioactive compounds and hormonal precursors, including sulfated polysaccharides (such as alginate, laminarin, and fucoidans), free amino acids, polyamines, vitamins, along with essential minerals and trace elements (Craigie, 2011; Chiaiese et al., 2018; Shukla et al., 2019). The synergistic interaction among these constituents confers seaweed extracts with emergent properties that go beyond the effects of individual molecules, characterizing them as functional complexes with broad and multifaceted impacts on plant physiology (Yakhin et al., 2017).

Several physiological, biochemical, and molecular studies have demonstrated that seaweed extracts act on multiple plant signaling pathways, positively modulating root growth and photosynthetic activity, promoting the accumulation of osmoprotectants (for example, proline and soluble sugars), enhancing the activity of antioxidant enzymes [superoxide dismutase (SOD), catalase (CAT), ascorbate peroxidase (APX)], and stimulating genes related to defense and secondary metabolism (Patel et al., 2018; Hernández-Herrera et al., 2022; Irani et al., 2025). These effects are particularly relevant under abiotic stress conditions such as drought, salinity, or extreme temperatures, where the application of seaweed extracts can mitigate physiological losses and help maintain agronomic performance across a wide range of crops (Battacharyya et al., 2015). Moreover, growing evidence suggests that these products also influence plant–microbe interactions by promoting beneficial changes in rhizosphere microbiota composition and in the expression of genes involved in biotic recognition and signaling (Shukla et al., 2019). In addition, *Ascophyllum nodosum* extracts have been shown to enhance endogenous phytohormone biosynthesis, increasing cytokinins and abscisic acid while reducing auxin levels, through transcriptional regulation of key biosynthetic (*IPT3–5*, *NCED3*, *ABA2*) and catabolic (*CKX4*) genes, thereby improving plant resilience to abiotic stress (Wally et al., 2013). This holistic interpretation aligns with the “functional plant health” concept proposed by Du Jardin et al. (2025), which redefines crop vitality as the dynamic ability to maintain physiological balance, respond to stress, and recover function under variable environments. In this context, seaweed extracts can be interpreted as preventive tools that strengthen the plant’s internal regulatory networks, contributing to resilience and stable productivity.

From an agronomic perspective, the positive effects of seaweed extracts have been documented in numerous crops, including vegetables, fruit trees, forage grasses, and cereals, with favorable responses in biomass production, nutrient accumulation, photosynthetic parameters, antioxidant metabolism, and yield (Battacharyya et al., 2015; Li et al., 2022). In a meta-analysis including more than one thousand experimental comparisons, Li et al. (2022) reported an average yield increase of 17.9% with the application of non-microbial biostimulants, with seaweed extracts representing one of the most prominent groups. However, although these results confirm their agronomic relevance, the available analyses still encompass multiple categories of biostimulants, limiting the precise understanding of the effects attributable exclusively to seaweed extracts. Seaweeds themselves represent a diverse group of multicellular marine macroalgae classified into three major groups: green algae (Chlorophyta), brown algae (Phaeophyceae), and red algae (Rhodophyta) (Deolu-Ajayi et al., 2022). This diversity is reflected in the wide range of species incorporated into biostimulant formulations. Each group, and its well-studied genera such as *Ulva* (green), *Ascophyllum*, *Laminaria*, and *Sargassum* (brown), and *Kappaphycus* and *Gracilaria* (red), exhibits distinct biochemical profiles (Khan et al., 2009; Deolu-Ajayi et al., 2022; Trivedi et al., 2023; Ribeiro et al., 2024).

The biological efficacy of seaweed-derived biostimulants is strongly influenced by the extraction process used to obtain bioactive compounds from algal biomass. The efficacy of these extracts depends on multiple factors, including the algal species used, the target crop, the timing of application, the edaphoclimatic conditions, and, critically, the extraction method (Craigie, 2011; Shukla et al., 2019). Techniques such as alkaline extraction, enzymatic hydrolysis, microwave-assisted extraction, or ultrasound-assisted extraction yield extracts with distinct biochemical profiles and, consequently, different bioactivities (Chemat et al., 2012; Matsuyama, 2018). Process parameters, particularly pressure, temperature, and solvent characteristics, are decisive in determining the biochemical profile of seaweed extracts. Intensive extraction conditions may degrade native phytohormones, polysaccharides, and phenolic compounds, thereby reducing the physiological potential of the product (Plaza et al., 2010; Barba et al., 2015). Conversely, carefully controlled extractions, such as those employing moderate temperatures or aqueous–alkaline systems, can preserve the molecular integrity of bioactive compounds while promoting the release or transformation of previously bound or inactive metabolites into bioavailable forms, thereby increasing chemical complexity and potentially enhancing biological activity (Craigie et al., 2008). In this context, conducting a systematic meta-analysis focused specifically on the agronomic effects of seaweed extracts is essential to consolidate existing knowledge, identify methodological gaps, and propose evidence-based guidelines for their efficient use in agriculture. Thus, this study undertakes a quantitative meta-analysis of the agronomic effects of seaweed extracts in agricultural crops, synthesizing findings from the international scientific literature. Specifically, we address the following questions: How do climatic conditions, stress environments, and trial types influence the agronomic effectiveness of seaweed extract–based biostimulants? How do soil properties, particularly phosphorus, potassium, pH, salinity, and organic matter, modulate crop responses? Which crop groups exhibit the strongest and most consistent yield or biomass responses? How do dose range, phenological stage, and growth phase influence plant response dynamics? To what extent do application methods, co-application with other inputs, and management practices determine efficacy? Finally, how do formulation type, extraction process, and algal species affect agronomic response and the reliability of seaweed extract performance across studies?

## 2 Material and methods

### 2.1 Data sources, search strategy, and study selection

The study aimed to answer the following research question framed under the PICOC framework: “In agricultural and greenhouse trials (Population), does the application of seaweed extract-based biostimulants (Intervention) compared to untreated controls or conventional treatments (Comparison) improve crop yield and biomass (Outcome) across diverse agronomic conditions (Context)?” (García-Peñalvo, 2022). To address this, a bibliographic search was conducted in January 2025 across three major databases, Scopus, PubMed and Web of Science, without geographical or temporal restrictions beyond that date.

The general query (“biostimulant” AND “seaweed” AND “extract”) AND LIMIT-TO (DOCTYPE, “ar”) was first applied, returning 4,764 records in total (Scopus 2,721; PubMed 896; Web of Science 1,157). The search was then refined using the more specific equation (“biostimulant” AND “crop” AND “seaweed” AND “extract” AND (yield OR biomass)) AND LIMIT-TO(DOCTYPE, “ar”), which yielded 247 records in total. The query syntax was adapted for PubMed and Web of Science to ensure consistency in retrieval across platforms.

In addition to database searches, manual citation searching of the reference list from the designated guide paper (Li et al., 2022) was conducted, identifying 82 additional records, of which 11 were excluded for insufficient information, leaving 71 studies eligible for screening.

**Figure 1:**
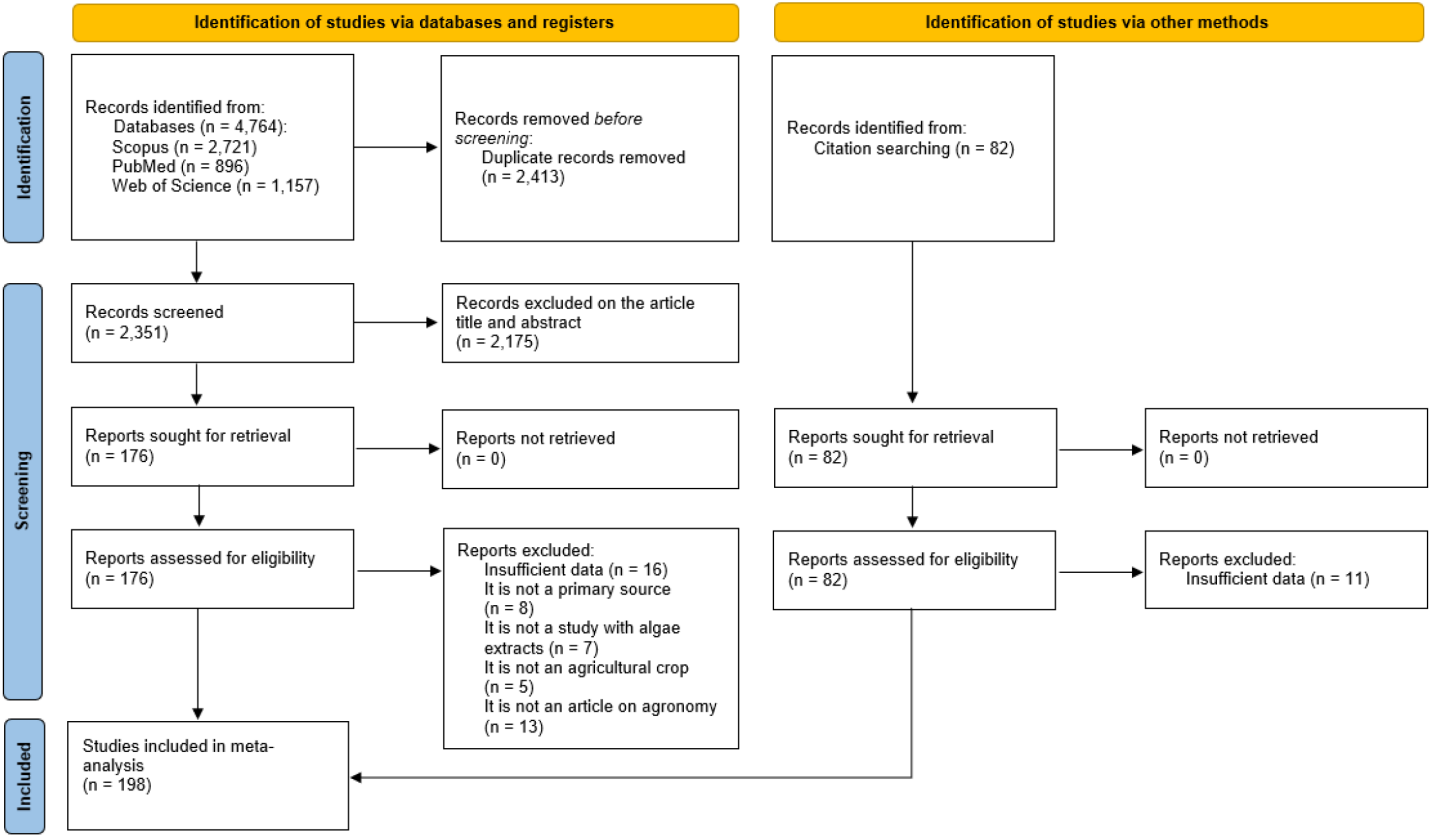
PRISMA 2020 flow diagram of study selection. Sources: Scopus, PubMed, Web of Science and citation searching; duplicates removed before screening. Boxes show counts (n) and reasons for exclusion. 198 studies were included.

The study selection process was conducted in accordance with the PRISMA 2020 guidelines and is summarized in the flow diagram presented in **Figure 1** (Page et al., 2021). This diagram illustrates the numbers of records identified, screened, assessed for eligibility and ultimately included in the meta-analysis (Haddaway et al., 2022).

### 2.2 Eligibility criteria

Field and greenhouse experiments using seaweed extract–based biostimulants that reported crop yield or biomass were included when data were expressed as means and standard deviations or as convertible error metrics together with sample sizes. Studies were excluded if they were *in vitro* or lacked yield/biomass data, if they were reviews or opinion pieces without primary data, if they evaluated non-seaweed-based biostimulants, or if they investigated systems outside the scope of agricultural crops.

### 2.3 Study selection process

An initial pool of 4,835 records (4,764 from databases and 71 from citation searching) was consolidated and deduplicated, yielding 2,422 unique records for title and abstract screening. Following independent screening by two reviewers, 2,175 records were excluded, and 247 full-text articles were assessed for eligibility. Of these, 49 studies were excluded, resulting in 198 studies included in the quantitative synthesis, comprising 2,021 independent treatment–control comparisons used for the meta-analysis (available in **Supplementary Materials S3**).

### 2.4 Data extraction

Data were extracted to address the moderators associated with each research question, encompassing climatic and stress conditions, soil properties, crop groups, phenological stages, application methods, formulations, and algal species. All extracted information was compiled into a standardized spreadsheet template by three independent reviewers, and any discrepancies were resolved by a fourth reviewer. Subsequently, seaweed extracts were classified according to their origin and physical formulation. Extracts were first categorized as either commercial or non-commercial: commercial extracts were defined as those with a trademark or explicitly described as commercial products in the source documents, whereas non-commercial extracts were experimental formulations or extracts prepared exclusively for research purposes without a registered trademark. In addition, extracts were classified by their physical formulation as either liquid or powder.

Crop species and categories were assigned according to the FAO guidelines for the agricultural census: cereals; vegetables and melons; fruits and nuts; oilseeds; roots and tubers; beverages and spices; legumes; sugar crops; and other crops (Food and Agriculture Organization of the United Nations, 2005).

Climatic conditions and water-regime classifications followed the Köppen–Geiger climate scheme: A (Af, Am, As, Aw); B (BWk, BWh, BSk, BSh); C (Cfa, Cfb, Csa, Csb, Cwa); and D (Dfa, Dfb, Dfc, Dsa) (Peel et al., 2007).

Soil properties were harmonized using standardized agronomic thresholds to reduce analytical and contextual heterogeneity across studies. Categorical criteria for pH, salinity (ECe), and available phosphorus and potassium followed Li et al. (2022), while soil texture and organic matter classes were assigned according to USDA guidelines (United States Department of Agriculture, N R C S, 2024a, 2024b). Nitrogen availability was not included among the harmonized soil variables because it was inconsistently reported across studies and measured in different analytical forms (e.g., nitrate, ammonium, or total N), which limited cross-study comparability; moreover, soil nitrogen pools are highly dynamic due to rapid microbial transformations (Robertson and Groffman, 2015). The classification thresholds used are shown in **Supplementary Materials S2**.

Reported application volumes (L ha^−1^) were converted to corresponding concentrations (mL L^−1^ or g L^−1^) for each crop using the calibration tables of Matthews et al. (2014); typical spray volumes ranged from 200 to 600 L ha^−1^ for field crops and approximately 1,000 L ha^−1^ for trees and shrubs. Plant phenological stages at the time of application were categorized according to the BBCH scale (Meier, 2001): 0, germination/sprouting/bud development; 1, leaf development (main shoot); 2, formation of lateral shoots/tillering; 3, stem elongation or rosette growth, shoot/cane development; 4, development of harvestable vegetative parts or propagation organs; 5, inflorescence emergence/heading; 6, flowering; 7, fruit development; 8, fruit and seed ripening/coloration; and 9, senescence or onset of dormancy.

Seaweed extract preparation methods were recorded as described by (Shukla et al., 2019): water-based extraction, acid hydrolysis, alkaline hydrolysis, microwave-assisted extraction, ultrasound-assisted extraction, enzyme-assisted extraction, supercritical fluid extraction, and pressurized liquid extraction.

Yield and biomass data were also extracted for alternative treatments to seaweed extracts reported within the same studies, which were classified as chitosan, humic and fulvic acids, protein hydrolysates, silicon, phosphates, plant extracts, moringa leaf extract, pesticides, fertilizers, microorganisms, organic amendments, and phytohormones, following the categories previously adopted by Li et al. (2022).

To provide a practical economic interpretation of the meta-analytic responses, two complementary FAOSTAT datasets (available in **Supplementary Materials S5**) were used: producer prices expressed in USD kg^−1^ and production quantity expressed in tons (Food and Agriculture Organization of the United Nations, 2024). Country-level producer prices were aggregated as production-weighted means for each crop using the corresponding production quantities for each country–crop combination, in order to obtain more representative crop-level price estimates and reduce the influence of extreme values from countries with marginal production, following the general FAO methodological principle that agricultural aggregate indicators should be calculated as weighted averages according to the relative contribution of each unit to total production value (Food and Agriculture Organization of the United Nations, 2013). Crop-level weighted prices were subsequently summarized across FAO crop groups and integrated with the meta-analytic yield responses of each group to compare the relationship between agronomic response and crop economic value across crop categories, consistent with comparative agricultural analyses that assess yield responses across different crop groups (de Ponti et al., 2012; Seufert et al., 2012).

Quantitative data extraction from figures and graphical elements was performed using ImageJ v1.53 (Schneider et al., 2012). The geographic distribution of the identified studies was visualized using ArcMap (Esri, 2020). When standard errors were not reported in the original studies, the standard deviation (SD) was estimated as a percentage based on the mean SD of the available studies within each crop category, following the procedure described by Schütz et al. (2018). The estimated SD values were as follows: root/tuber 7.8%, cereals 4.6%, fruit and nuts 6.9%, leguminous 3.9%, oilseed 4.1%, other crops 4.0%, sugar crops 5.5%, and vegetables and melons 6.4%.

All extracted variables were subsequently used as moderators in the meta-analytic models to quantify their contribution to the variability in yield and biomass responses across studies.

### 2.5 Quality assessment and risk of bias

To address the presence of extreme values that could potentially bias statistical analyses, a robust outlier treatment procedure was implemented, combining logarithmic transformation and winsorization. Prior to outlier detection, all continuous variables were log-transformed to normalize their distributions and reduce the influence of extreme values on the scale. Outliers were then identified using the interquartile range (IQR) method, whereby values falling below Q1 − 1.5 × IQR or above Q3 + 1.5 × IQR in the log-transformed space were classified as outliers. Rather than excluding these observations, winsorization was applied by clipping outlying values to the respective threshold boundaries, thereby preserving sample size while mitigating the disproportionate influence of extreme observations. This approach ensured that the statistical properties of the transformed data were maintained while reducing the potential for outliers to distort subsequent meta-analytic computations. Studies containing only one observation were excluded from the analysis.

Given that several moderators showed a substantial proportion of missing data (>50%), the association between the missing-data pattern and the study of origin was first evaluated before choosing between data imputation and complete-case analysis. Specifically, a χ^2^ test of independence (H_0_: no association between paper of origin and missingness; H_1_: an association exists) was performed, with the test statistic following a chi-square distribution and degrees of freedom equal to (number of studies − 1) × (number of missingness categories − 1). A significant result (*p* < 0.05) would indicate that data are not missing completely at random (MCAR). Based on the test outcomes, complete-case analysis was adopted rather than imputation, as imputing under non-random missingness could introduce bias into the meta-analytic estimates.

Publication bias and small−study effects were assessed by visual inspection of funnel plots (Sterne and Egger, 2001), plotting each study’s log response ratio against its standard error, and by Egger’s regression test (Egger et al., 1997), which regressed standardized effect sizes on their standard errors under a random−effects model; a p−value < 0.05 was taken as evidence of asymmetry.

### 2.6 Data synthesis and statistical analysis

A total of 2,021 treatment-control data pairs were initially extracted from 198 studies. To ensure the mathematical validity of the log-transformation, any observations involving zero or negative mean values were excluded prior to analysis. To address within-study dependence and prevent the statistical errors associated with pseudo-replication, raw observations were systematically aggregated using the aggregate function in the metafor package for R (Viechtbauer, 2010). For the global effect analysis, data were collapsed into study-level units. For moderator analyses, observations were aggregated into unique study × moderator category combinations, resulting in 195 independent study-level units for the global model. Because aggregation was performed prior to model fitting, no study contributed multiple dependent effect sizes to the same statistical model, thereby satisfying the independence assumption required for conventional random-effects meta-analysis. Meta-analysis was conducted using random-effects models to account for between-study heterogeneity. Effect sizes were calculated as log response ratios (LRR = ln[treatment mean/control mean]), and their variances were estimated via the delta method, incorporating group variances and sample sizes. The overall effect and between-study variance (τ^2^) were estimated using the restricted maximum likelihood (REML) method (Patterson and Thompson, 1971), with each independent unit weighted by the inverse of its LRR variance. Heterogeneity was quantified using Cochran’s Q and I^2^ statistics. Results are reported as LRR with 95% confidence intervals (CI) and back-transformed to percentage change using the formula:% change = (e^LRR – 1) × 100. Forest plots visualize individual and pooled estimates. Publication bias was rigorously evaluated through visual inspection of funnel plots and the formal Egger’s linear regression test. When significant asymmetry was detected, the Trim-and-Fill method was employed to estimate the number of potentially missing studies and to calculate a bias-adjusted pooled effect size.

All statistical analyses were conducted in R (v4.4.3). Meta-analytic models were fitted with the metafor package (v4.8-0) (Viechtbauer, 2010), data manipulation and aggregation used dplyr (v1.1.4), and Excel files were imported via readxl (v1.4.5). Figures were generated with base R plotting functions.

All data sources and detailed statistical outputs are provided in the **Supplementary Materials S3**, **S4**, to ensure full transparency and reproducibility of the meta-analytic procedures.

## 3 Results

### 3.1 Climate, stress, and type of trial

**Figure 2:**
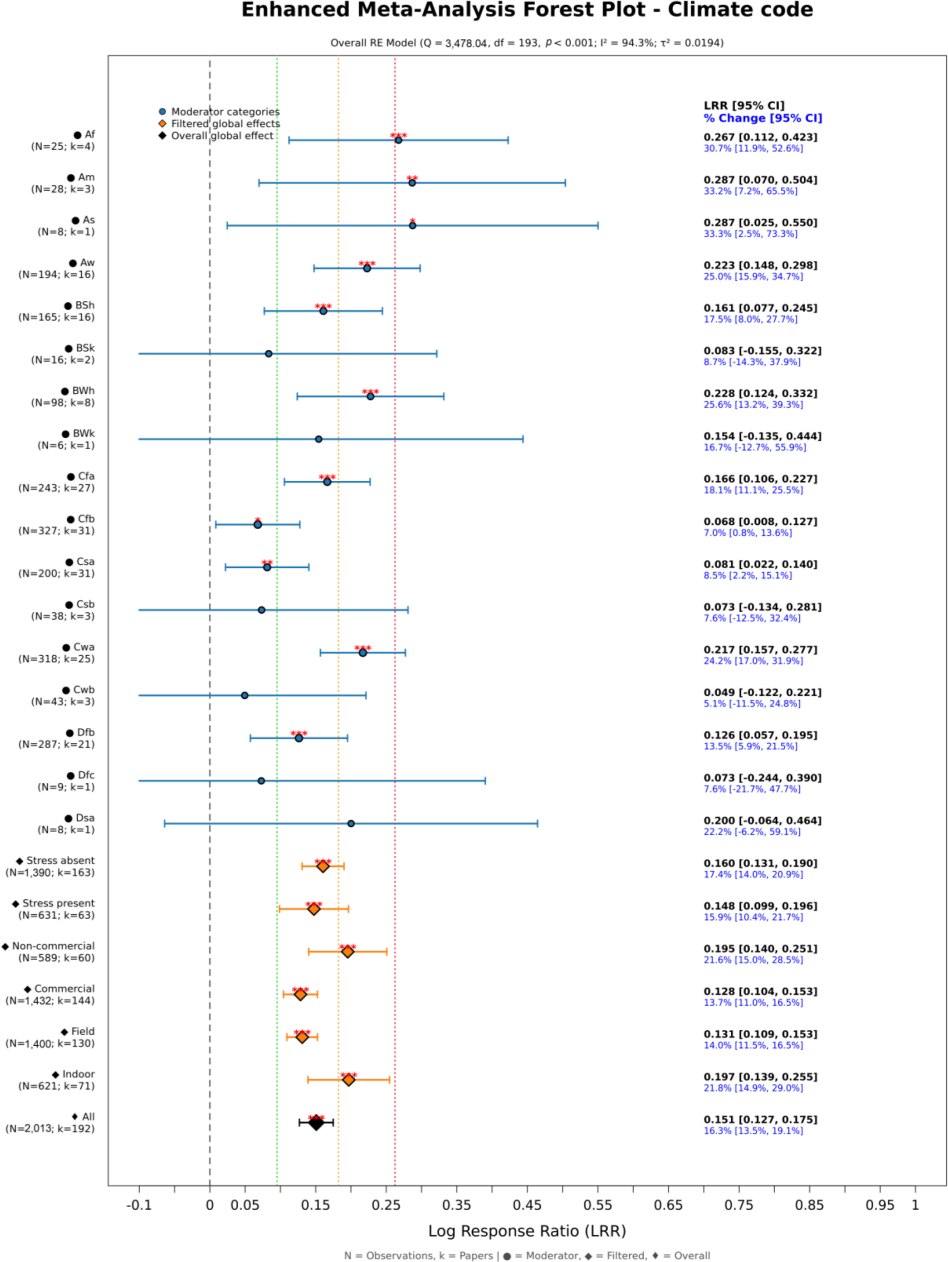
Yield effects expressed as log response ratio (LRR), stratified by Köppen–Geiger climates and other moderators. Points show subgroup means with 95% confidence intervals (CI); diamonds are random-effects pooled estimates; the vertical line marks LRR = 0 (no effect). Asterisks on diamonds indicate significance (*p < 0.05, **p < 0.01, ***p < 0.001). Sample sizes (N observations; k papers) appear next to labels.

**Figure 3:**
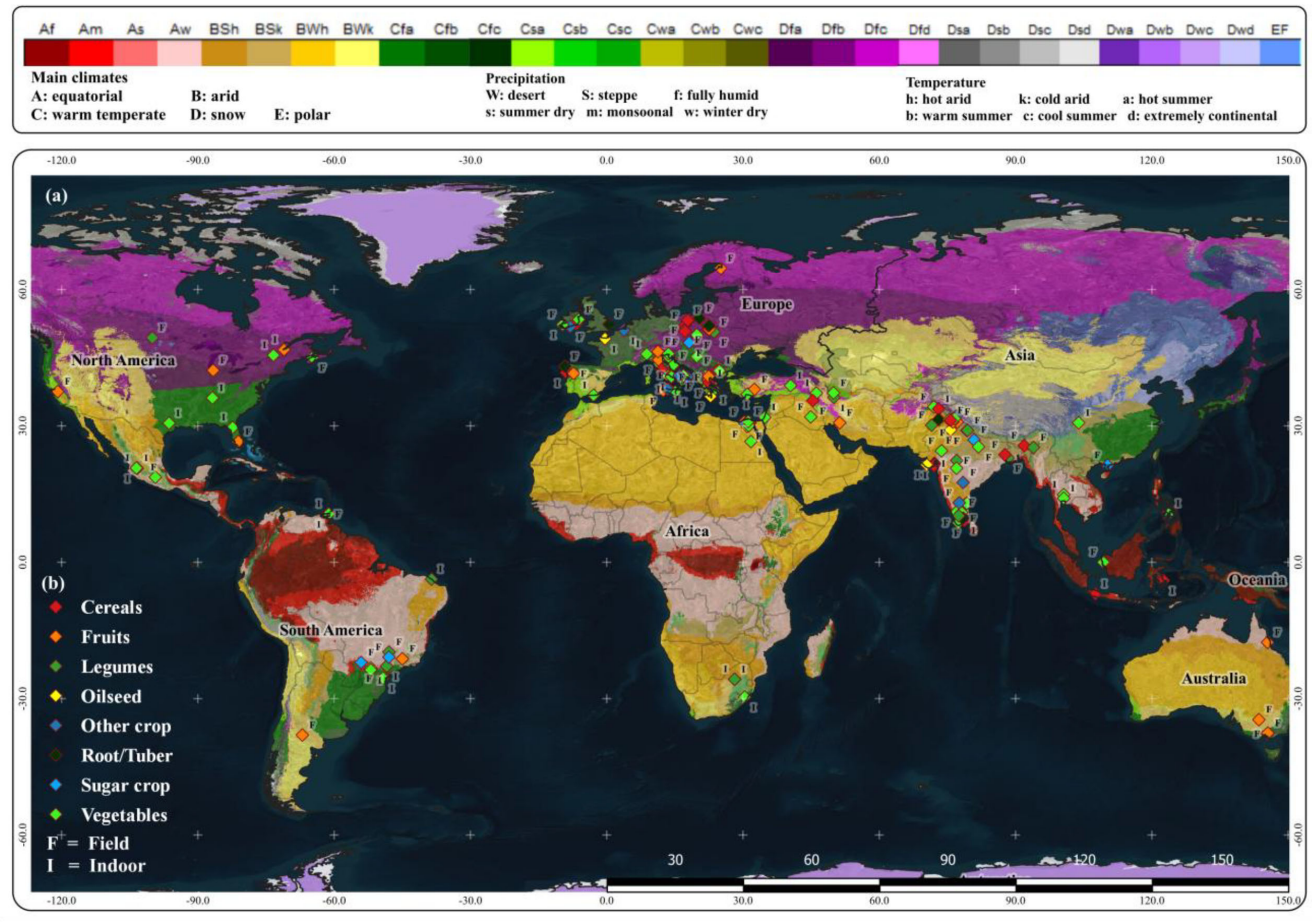
Global distribution of included trials overlaid on Köppen–Geiger climate classes (Esri, 2009). **(a)** Study-site locations. **(b)** Crop-group key; symbol color encodes crop group and the letter marks setting (F, field; I, indoor). Counts: field N = 130, indoor N = 69.

The overall yield effect was positive, averaging 16.3% (CI 13.5–19.1%) (**Figure 2**). Comparable responses were observed under non-stress conditions (17.4% [CI 14.0–20.9%]) and stress conditions (15.9% [CI 10.4–21.7%]). Controlled-environment experiments showed effects of 21.8% (CI 14.9–29.0%), while field trials exhibited positive responses of 14.0% (CI 11.5–16.5%). Studies using non-commercial extracts reported responses of 21.6% (CI 15.0–28.5%), compared with 13.7% (CI 11.0–16.5%) for commercial extracts. Across Köppen–Geiger climate zones, the largest effects were observed in tropical climates, including monsoon Am and savannah As/Aw regions (approximately 30–33%, with confidence intervals fully positive), followed by warm arid BWh (25.6% [CI 13.2–39.3%]) and warm semi-arid BSh zones (17.5% [CI 8.0–27.7%]), whereas effects in cooler zones with limited representation were not statistically significant. The geographical distribution (**Figure 3**) indicates a concentration of trials in temperate Europe and tropical-monsoon South/Southeast Asia, with fewer studies in the Americas, Africa, and Australia and minimal representation in boreal or polar regions.

### 3.2 Soil conditions as modulators

Soil-related moderators were summarized in three result tables covering fertility status (**Table 1**), soil pH and salinity (**Table 2**), and soil physical condition (**Table 3**).

**Table 1:** Yield responses to seaweed-based biostimulants across fertility status.

Soil property/classification	N	k	LRR	95% CI	% Change	Sig.
Soil P content						
Very low	382	39	0.159	[0.120, 0.197]	17.2	***
Low	79	9	0.152	[0.077, 0.227]	16.4	***
Medium	45	4	0.093	[-0.005, 0.190]	9.7	ns
Optimal	576	47	0.119	[0.085, 0.152]	12.6	***
All (P)	1,085	98	0.135	[0.112, 0.159]	14.5	***
Soil K content						
Very low	65	8	0.137	[0.049, 0.225]	14.7	**
Low	94	9	0.118	[0.044, 0.196]	12.5	**
Medium	255	21	0.152	[0.102, 0.202]	16.5	***
Optimal	663	60	0.131	[0.099, 0.162]	14.0	***
All (K)	1,077	97	0.135	[0.111, 0.158]	14.4	***
Soil organic matter (SOM)						
Very low	89	10	0.124	[0.059, 0.190]	13.2	***
Low	219	26	0.122	[0.081, 0.164]	13.0	***
Medium	391	24	0.151	[0.107, 0.196]	16.3	***
High	207	21	0.13	[0.071, 0.189]	13.9	***
All (SOM)	906	75	0.133	[0.108, 0.157]	14.2	***

Effect sizes are reported as log response ratios (LRR) with 95% confidence intervals (CI) from random-effects models (REML). *N* is the number of observations and *k* the number of papers. Significance (Sig.): p < 0.01 **, p < 0.001 ***; ns, not significant. Global random-effects models showed substantial heterogeneity for soil phosphorus (Q = 1,041.03, df = 98, *p* < 0.001; I^2^ = 89.4%; τ^2^ = 0.0084), potassium (Q = 1,039.33, df = 97, *p* < 0.001; I^2^ = 89.5%; τ^2^ = 0.0085), and organic matter (Q = 933.68, df = 80, *p* < 0.001; I^2^ = 89.0%; τ^2^ = 0.0073). Classes are ordered from lower to higher nutrient or organic matter levels.

**Table 2:** Yield responses to seaweed-based biostimulants across soil pH and electrical conductivity classes.

Soil property/classification	N	k	LRR	95% CI	% Change	Sig.
Soil pH						
Strongly acid	285	17	0.129	[0.073, 0.185]	13.8	***
Moderately acid	165	13	0.107	[0.040, 0.175]	11.3	**
Slightly acid	88	14	0.137	[0.070, 0.203]	14.6	***
Neutral	362	35	0.137	[0.091, 0.183]	14.7	***
Moderately alkaline	302	25	0.171	[0.123, 0.219]	18.7	***
Strongly alkaline	26	5	0.123	[0.024, 0.222]	13.1	*
All (pH)	1,228	105	0.14	[0.116, 0.163]	15.0	***
Electrical conductivity (CE)						
Nonsaline	476	49	0.154	[0.117, 0.191]	16.7	***
Slightly saline	47	6	0.235	[0.119, 0.351]	26.5	***
Moderately saline	22	2	0.258	[0.078, 0.438]	29.4	**
Strongly saline	4	1	0.168	[-0.299, 0.636]	18.3	ns
All (CE)	549	58	0.166	[0.130, 0.201]	18.0	***

Effect sizes are reported as log response ratios (LRR) with 95% confidence intervals (CI) from random-effects models (REML). *N* is the number of observations and *k* the number of papers. Significance (Sig.): *p* < 0.05 *, p < 0.01 **, p < 0.001 ***; ns, not significant. Global random-effects models showed substantial heterogeneity for soil pH (Q = 1,272.87, df = 108, *p* < 0.001; I^2^ = 92.1%; τ^2^ = 0.0104) and soil salinity (electrical conductivity) (Q = 309.68, df = 57, *p* < 0.001; I^2^ = 85.4%; τ^2^ = 0.0107). Soil pH classes are ordered from strongly acidic to strongly alkaline, and electrical conductivity classes from non-saline to strongly saline.

**Table 3:** Yield responses to seaweed-based biostimulants across soil texture classes.

Soil texture	N	k	LRR	95% CI	% Change	Sig.
Clay	198	14	0.167	[0.095, 0.238]	18.1	***
Clay loam	109	18	0.224	[0.145, 0.302]	25.1	***
Fine sand	9	1	0.073	[-0.220, 0.365]	7.6	ns
Fine sandy loam	16	1	0.033	[-0.194, 0.261]	3.4	ns
Loamy	228	16	0.118	[0.055, 0.181]	12.5	***
Loamy sand	104	8	0.228	[0.108, 0.348]	25.7	***
Sandy	104	11	0.113	[0.020, 0.205]	11.9	*
Sandy clay loam	30	5	0.176	[0.041, 0.310]	19.2	*
Sandy loam	237	25	0.121	[0.062, 0.179]	12.8	***
Silt loam	87	8	0.144	[0.059, 0.229]	15.5	***
Silty clay	38	3	0.136	[-0.032, 0.303]	14.5	ns
Silty clay loam	28	3	0.171	[-0.084, 0.426]	18.7	ns
All (overall)	1,188	111	0.148	[0.121, 0.174]	15.9	***

Effect sizes are reported as log response ratios (LRR) with 95% confidence intervals (CI) from random-effects models (REML). *N* is the number of observations and *k* the number of papers. Significance (Sig.): *p* < 0.05 *, p < 0.001 ***; ns, not significant. The global random-effects model showed substantial heterogeneity (Q = 1,156.84, df = 112, *p* < 0.001; I^2^ = 90.7%; τ^2^ = 0.0129).

Soil fertility classes showed relatively consistent yield effects, with responses generally clustering around 15–17% and no clear trend across fertility gradients. For soil phosphorus (P), positive effects were observed under very low and low fertility conditions (approximately 16–17%), whereas the medium P class did not differ significantly; optimal P levels showed moderate positive responses. Potassium (K) classes exhibited uniformly positive effects across the fertility gradient, with yield responses centered around 13–17% and overlapping confidence intervals among categories (**Table 1**).

Soil organic matter (SOM) classes showed yield effects of similar magnitude, with responses generally clustering around 13–16% and overlapping confidence intervals across categories, without a clear linear pattern. The overall pooled effect was positive (approximately 14%), and no SOM class exhibited a distinctly divergent response (**Table 1**).

Soil pH categories showed positive yield effects of comparable magnitude, with responses generally clustering around 11–19% and overlapping confidence intervals across classes, yielding an overall effect of approximately 15%. Soil electrical conductivity (EC) classes displayed positive responses from non-saline to moderately saline conditions, reaching increases of up to 25–30%, whereas the strongly saline class did not exhibit a significant response (**Table 2**).

Meta-regression analyses using continuous soil fertility variables (extractable P and K, soil organic matter, pH, and electrical conductivity) did not reveal consistent linear relationships with yield responses expressed as log response ratios. Regression slopes were generally small and associated with wide confidence intervals, indicating limited explanatory power for between-study variability. Accordingly, the corresponding meta-regression plots are provided in the **Supplementary Materials S1**.

Soil texture classes showed consistently positive yield effects, with overlapping confidence intervals across most categories and no single texture exhibiting a distinctly divergent response. Yield increases generally clustered below 20%, although some fine and medium textured soils reached values of approximately 25%, notably in clay loam and loamy sand classes (**Table 3**).

### 3.3 Response by crop group

**Figure 4:**
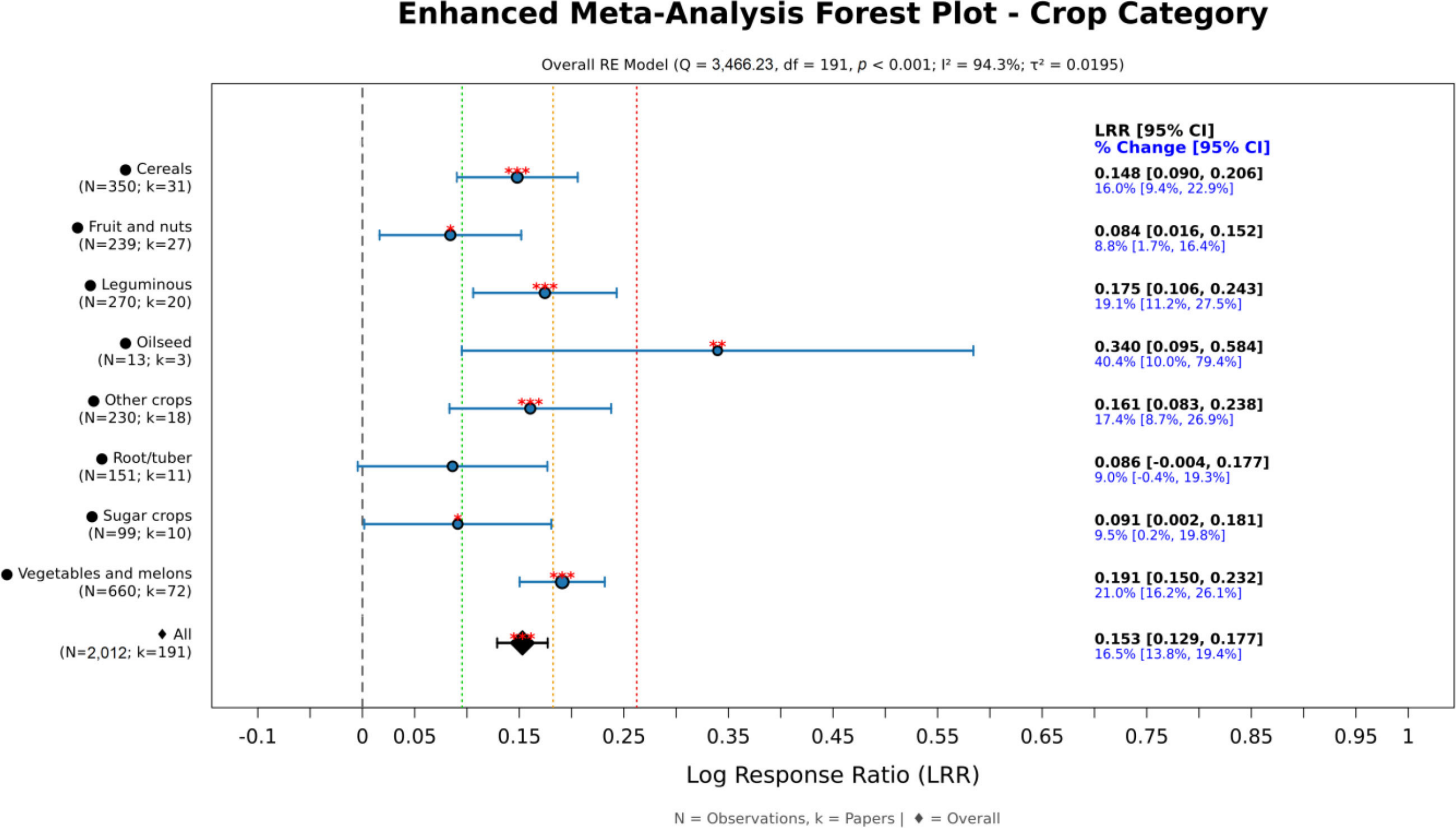
Yield effects expressed as log response ratio (LRR) by crop category. Points show subgroup means with 95% confidence intervals (CI); the vertical line marks LRR = 0. Right-hand labels report LRR and % change. Asterisks denote significance (*p < 0.05, **p < 0.01, ***p < 0.001). Sample sizes (N observations; k papers) appear next to labels.

Yield effects varied among crop groups (**Figure 4**). The largest responses were observed in vegetables and melons (around 20%), followed by legumes and cereals (approximately 16–19%). Fruit trees and nuts, as well as sugar crops, showed more modest increases (around 9–10%), whereas responses in roots and tubers were not statistically significant. Oilseeds exhibited a high point estimate (around 40%), accompanied by wide confidence intervals reflecting limited representation.

#### 3.3.1 Economic interpretation of yield effects

**Figure 5:**
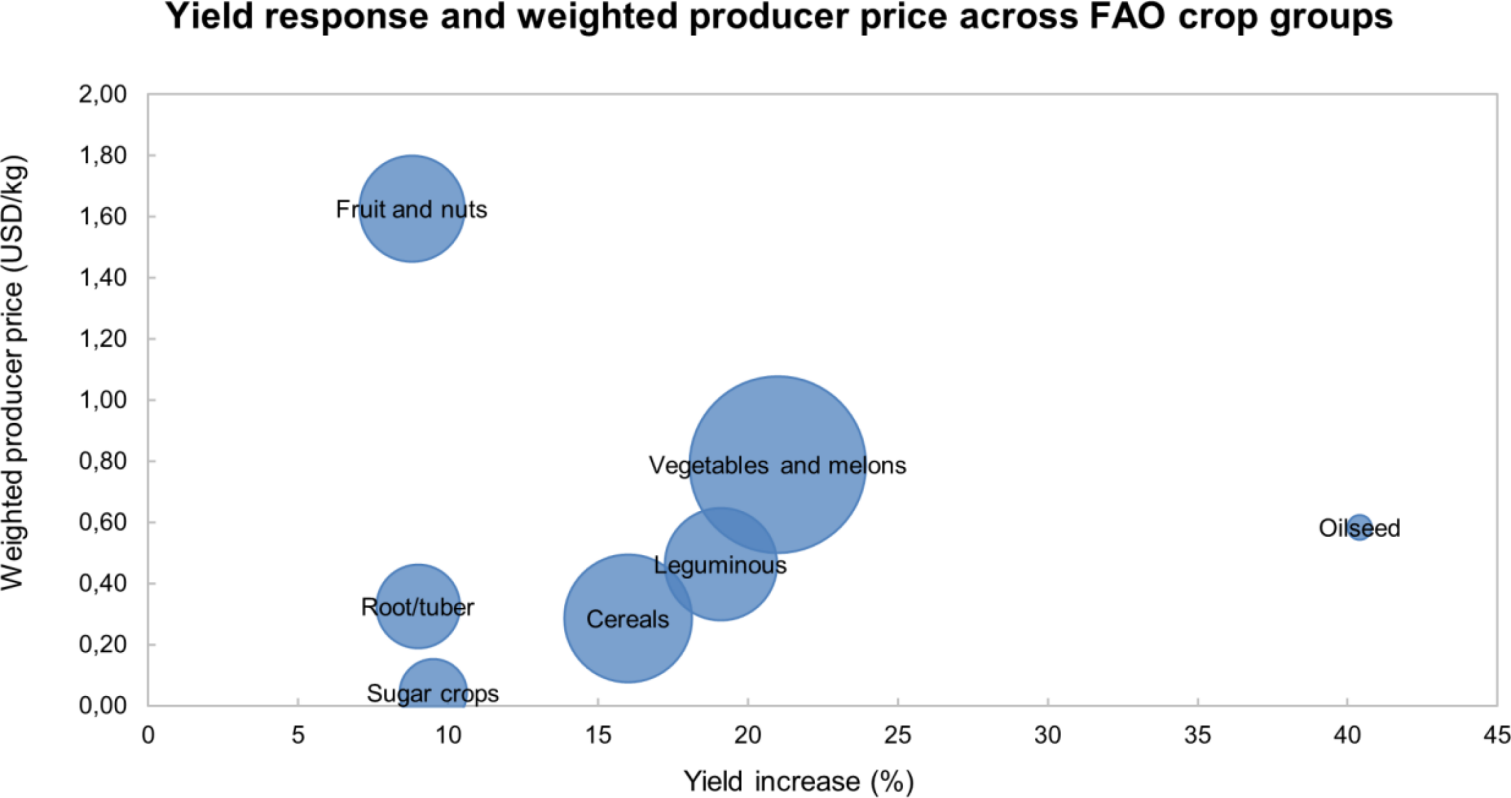
Yield response and weighted producer price across FAO crop groups. The horizontal axis shows the meta-analytic yield response expressed as percentage change relative to the untreated control, and the vertical axis shows the production-weighted producer price (USD kg^−1^) calculated from FAOSTAT data for each crop group. Bubble size is proportional to the number of observations included in each crop group.

When yield responses were interpreted together with production-weighted producer prices (**Figure 5**), clear differences emerged among FAO crop groups in their potential economic relevance. Vegetables and melons showed the most favorable overall profile, combining a strong yield increase (21%) with a relatively high weighted price (0.79 USD kg^−1^) and the largest evidence base (N = 660). Fruit and nuts had the highest weighted price (1.63 USD kg^−1^), indicating that even a smaller average yield gain (9%) may still translate into substantial economic benefits in high-value crops. Leguminous crops also showed a favorable pattern, with a strong yield response (19%) and an intermediate weighted price (0.46 USD kg^−1^). Cereals showed a consistent positive response (16%), but their lower weighted price (0.29 USD kg^−1^) suggests a more moderate economic magnitude. Oilseed crops had the largest numerical yield increase (40%), although this estimate was supported by a limited number of observations (N = 13) and should therefore be interpreted cautiously. In contrast, root/tuber and sugar crops combined smaller yield gains (9–10%) with comparatively low weighted prices (0.32 and 0.04 USD kg^−1^, respectively), indicating lower economic potential.

### 3.4 Dose-response, operating range, and phenology

**Figure 6:**
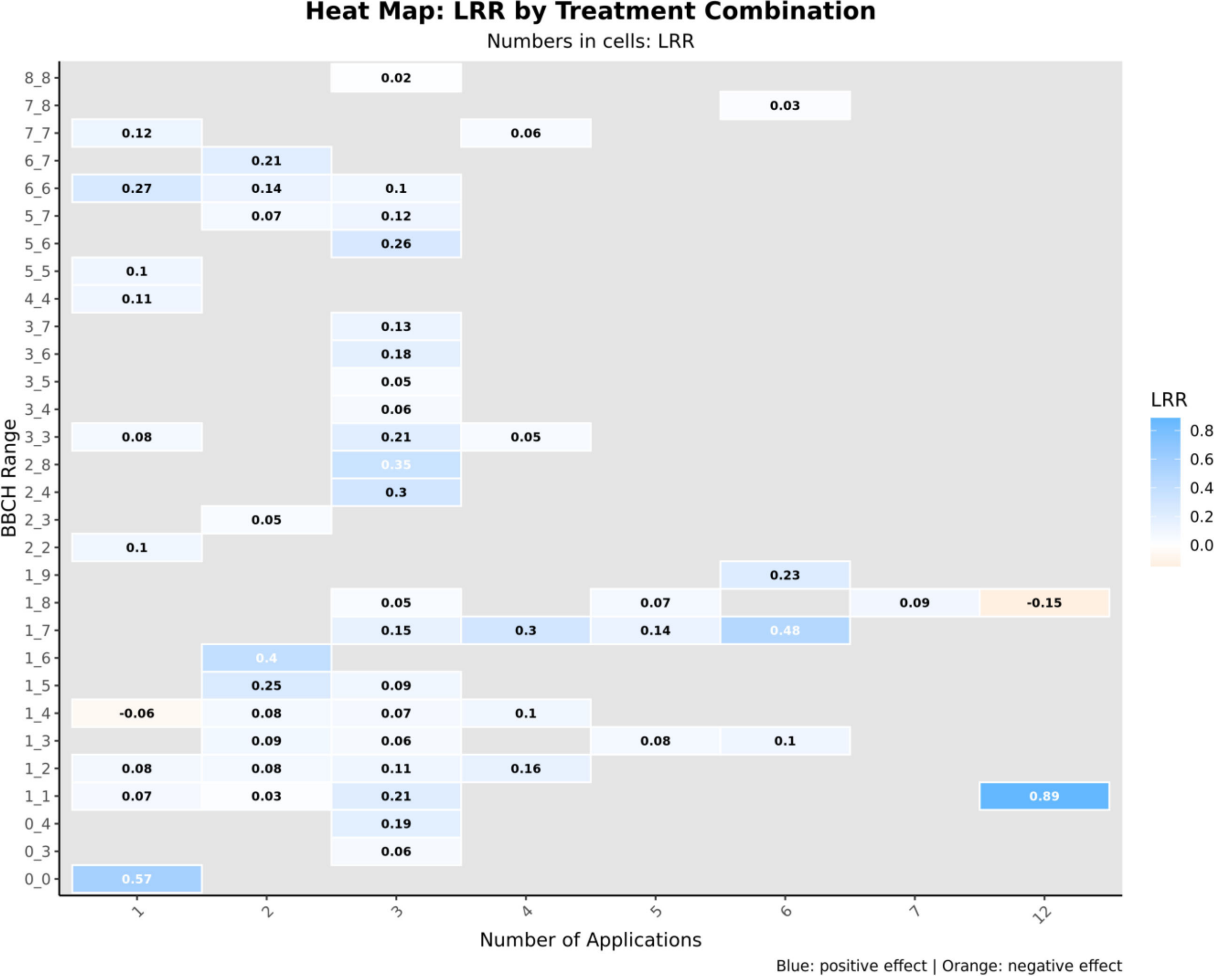
Yield response (LRR) across BBCH growth stages and number of applications. BBCH phenological stage (y-axis) and number of applications (x-axis). BBCH stages are defined as follows: 0, germination, sprouting, or bud development; 1, leaf development (main shoot); 2, formation of lateral shoots or tillering; 3, stem elongation or rosette growth, shoot or cane development; 4, development of harvestable vegetative parts or propagation organs; 5, inflorescence emergence or heading; 6, flowering; 7, fruit development; 8, fruit and seed ripening or coloration; and 9, senescence or onset of dormancy. Values within cells represent LRR, with blue shading indicating stronger positive responses.

Dose meta-regression did not identify a significant linear relationship between applied concentration and yield response (**Supplementary Materials S1**). The BBCH-by-number-of-applications heatmap showed that early programs (BBCH 1–3) combined with 4–6 applications concentrated the most consistent positive responses, with yield increases commonly around 26–42% (**Figure 6**). Higher responses were observed in isolated treatment combinations, reaching increases of approximately 80–150%, while occasional negative responses (around –10%) were also detected in specific configurations.

### 3.5 Method of application, co-application, and alternative treatments

Among application methods, soil-directed approaches showed the greatest yield increases, with fertigation and drench applications reaching approximately 19–21%, exceeding foliar application (15%) and combined programs (14%). Seed treatment did not show a significant response. Co-application with other inputs resulted in yield increases of around 4% relative to the biostimulant applied alone. When alternative inputs were compared with seaweed extracts, only plant extracts showed a significant positive response (14%), whereas the remaining categories exhibited responses close to zero or high uncertainty, with confidence intervals overlapping null effects (**Table 4**).

**Table 4:** Yield responses to seaweed-based biostimulants across application strategies and alternative or complementary treatments.

Classification/moderator	N	k	LRR	95% CI	% Change	Sig.
Application method (AP)						
Drench (D)	232	28	0.174	[0.101, 0.247]	19.0	***
Fertigation (R)	132	16	0.187	[0.082, 0.293]	20.6	***
Foliar (F)	1,435	142	0.144	[0.115, 0.173]	15.5	***
Program (D+R+F)	200	16	0.133	[0.044, 0.221]	14.2	**
Seed treatment	14	2	0.107	[-0.143, 0.358]	11.3	ns
Overall (AP)	2,013	191	0.149	[0.124, 0.174]	16.0	***
Co-application (CA)						
Applied alone	886	67	0.144	[0.105, 0.183]	15.5	***
Applied with another product	314	23	0.175	[0.141, 0.209]	19.1	***
Overall (CA)	1,200	90	0.156	[0.126, 0.187]	16.9	***
Alternative treatments vs seaweed extracts (AT)						
Chitosan	17	5	-0.007	[-0.121, 0.108]	-0.7	ns
Fertilizers	202	26	0.072	[0.010, 0.133]	7.4	*
Humic and fulvic acids	61	13	-0.009	[-0.094, 0.077]	-0.9	ns
Microorganisms	60	11	0.009	[-0.105, 0.123]	0.9	ns
Moringa leaf extract	5	1	0.128	[-0.184, 0.439]	13.6	ns
Organic amendments	50	7	0.002	[-0.105, 0.110]	0.2	ns
Pesticides	16	4	0.07	[-0.082, 0.222]	7.3	ns
Phytohormones	73	11	-0.026	[-0.122, 0.069]	-2.6	ns
Plant extracts	22	7	0.135	[0.031, 0.239]	14.4	*
Protein hydrolysates	147	27	0.023	[-0.033, 0.080]	2.4	ns
Silicons	6	3	-0.126	[-0.296, 0.044]	-11.8	ns
Overall (AT)	659	74	0.028	[-0.000, 0.057]	2.9	ns

Effect sizes are reported as log response ratios (LRR) with 95% confidence intervals (CI) from random-effects models (REML). *N* is the number of observations and *k* the number of papers. Significance (Sig.): *p* < 0.05 *, *p* < 0.01 **, *p* < 0.001 ***; ns, not significant. Global random-effects models showed substantial heterogeneity across application strategies and complementary treatment analyses. For the seaweed extract application method, (Q = 4,092.93, df = 203, *p* < 0.001; I^2^ = 94.9%; τ^2^ = 0.0227). For application alone versus in combination with other products, (Q = 8,406.02, df = 205, *p* < 0.001; I^2^ = 95.4%; τ^2^ = 0.0245). For alternative or complementary treatments applied alongside seaweed-based biostimulants, (Q = 1,076.22, df = 114, *p* < 0.001; I^2^ = 93.3%; τ^2^ = 0.0157).

### 3.6 Formulation and extraction method

Although powder formulations showed numerically higher yield responses than liquid formulations (22.6% vs. 15.3%, respectively), the between-group difference was not statistically significant (QM(df = 1) = 2.38, *p* = 0.123). This indicates that formulation type did not significantly explain variability in yield responses across studies. Liquid formulations accounted for the majority of observations (N = 1,714; k = 173) and displayed narrower confidence intervals, likely reflecting their greater representation in the dataset. In contrast, powdered seaweed formulations were represented by fewer studies (N = 299; k = 22) (**Table 5**), which may partly explain the wider confidence intervals associated with this category. Consequently, although the pooled estimate for powder formulations was higher, this apparent difference should be interpreted cautiously.

**Table 5:** Yield responses to seaweed-based biostimulants across product formulation and extraction method.

Category	N	k	LRR	95% CI	% Change	Sig.
Formulation						
Liquid	1,714	173	0.142	[0.117, 0.167]	15.3	***
Powder	299	22	0.203	[0.130, 0.277]	22.6	***
Overall	2,013	191	0.149	[0.125, 0.173]	16.0	***
Extraction method						
Acid hydrolysis	14	3	0.101	[-0.110, 0.312]	10.6	ns
Alkaline hydrolysis	410	47	0.192	[0.136, 0.248]	21.2	***
Complex enzymatic hydrolysis	3	1	0.042	[-0.025, 0.340]	4.3	ns
Enzyme-assisted extraction	134	15	0.009	[-0.078, 0.096]	0.9	ns
Pressurized liquid extraction	6	1	0.114	[-0.870, 1.097]	12.0	ns
Supercritical fluid extraction	12	1	-0.010	[-0.307, 0.286]	-1.0	ns
Ultrasound-assisted extraction	12	1	0.246	[-0.099, 0.590]	27.9	ns
Water-based extraction	1,352	123	0.135	[0.153, 0.185]	16.6	***
Overall	1,933	184	0.147	[0.120, 0.173]	15.8	***

Effect sizes are reported as log response ratios (LRR) with 95% confidence intervals (CI) from random-effects models (REML). *N* is the number of observations and *k* the number of papers. Significance (Sig.): *p* < 0.001 ***; ns, not significant. Global random-effects models showed substantial heterogeneity. For the seaweed extract product formulation, (Q = 3,468.16, df = 194, *p* < 0.001; I^2^ = 94.2%; τ^2^ = 0.0195). For seaweed extraction method, (Q = 4,292.02, df = 191, *p* < 0.001; I^2^ = 95.9%; τ^2^ = 0.0240).

Extraction method further differentiated yield responses (**Table 5**). Alkaline hydrolysis showed the largest and most consistently supported effects, with yield increases of approximately 20–21%, followed by aqueous extraction, which exhibited responses around 16–17%. Other extraction techniques yielded smaller point estimates or effects compatible with null responses, frequently accompanied by wide confidence intervals.

### 3.7 Species of algae

Species-level analyses revealed marked variability in yield responses across taxa (**Table 6**). Several species exhibited positive effects in the range of 16–28%, whereas others showed smaller responses (around 7%) or effects compatible with null values. *Ascophyllum nodosum* displayed a stable response of approximately 16%, closely aligned with the overall species-level estimate and representing the most precisely estimated effect due to its larger evidence base.

**Table 6:** Yield responses to seaweed-based biostimulants across algae species .

Category	N	k	LRR	95% CI	% Change	Sig.
Seaweed extracts by species (general)						
*Ascophyllum nodosum*	873	95	0.151	[0.115, 0.188]	16.3	***
*Ecklonia maxima*	380	36	0.065	[0.010, 0.119]	6.7	*
*Fucus* spp.	14	2	0.226	[-0.060, 0.513]	25.4	ns
*Gracilaria* spp.	76	12	0.223	[0.134, 0.312]	24.9	***
*Kappaphycus* sp.	228	25	0.176	[0.111, 0.241]	19.2	***
*Laminaria* spp.	38	4	0.249	[0.054, 0.444]	27.8	*
*Padina* spp.	19	3	0.692	[0.440, 0.944]	99.8	***
*Saccharina* spp.	26	2	-0.018	[-0.256, 0.220]	-1.8	ns
*Sargassum* spp.	132	12	0.166	[0.068, 0.2264]	18.1	**
*Scenedesmus* spp.	3	1	0.484	[0.093, 0.875]	62.3	*
*Turbinaria* spp.	18	2	0.182	[-0.060, 0.425]	20.0	ns
*Ulva* spp.	38	3	0.201	[0.016, 0.387]	22.3	*
Overall	1,845	175	0.153	[0.126, 0.180]	16.5	***
Only commercial seaweed extracts						
*Ascophyllum nodosum*	786	85	0.143	[0.107, 0.178]	15.3	***
*Ecklonia maxima*	380	36	0.064	[0.014, 0.114]	6.6	*
*Gracilaria* spp.	39	5	0.141	[0.024, 0.259]	15.2	*
*Kappaphycus* sp.	122	12	0.187	[0.102, 0.271]	20.5	***
*Laminaria* spp.	38	4	0.253	[0.072, 0.434]	28.8	*
*Sargassum* spp.	39	4	0.059	[-0.082, 0.199]	6.1	ns
Overall	1,404	134	0.125	[0.099, 0.152]	13.4	***

Effect sizes are reported as log response ratios (LRR) with 95% confidence intervals (CI) from random-effects models (REML). *N* is the number of observations and *k* the number of papers. Significance (Sig.): p < 0.05 *, *p* < 0.01 **, *p* < 0.001 ***; ns, not significant. Global random-effects models showed substantial heterogeneity. For the seaweed extracts by species (general), (Q = 2,942.25, df = 145, *p* < 0.001; I^2^ = 94.8%; τ^2^ = 0.0183). For only commercial seaweed extracts, (Q = 4,338.50, df = 196, *p* < 0.001; I^2^ = 95.8%; τ^2^ = 0.0259).

When analyses were restricted to commercial extracts, the pooled yield response was approximately 13% (**Table 6**). Within this subset, *A. nodosum* contributed the largest number of observations and showed yield increases of around 15%, whereas other species exhibited either higher point estimates with wider confidence intervals or smaller, less consistent responses (**Table 6**).

**Figure 7:**
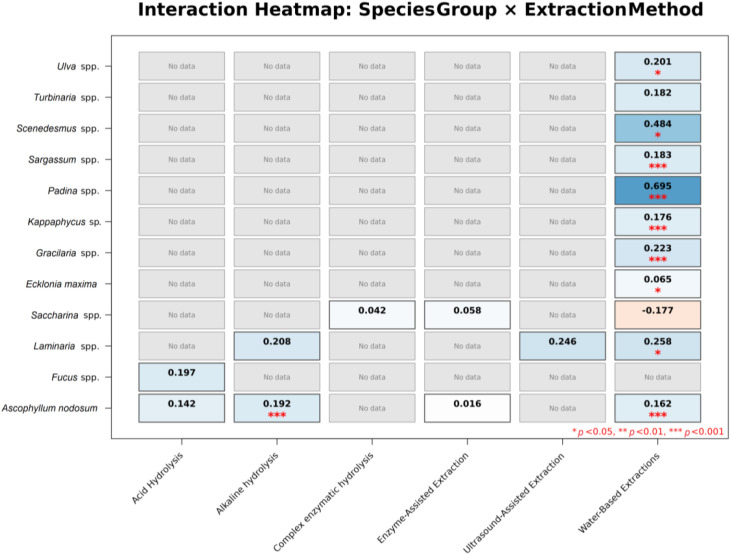
Interaction heatmap for species-by-extraction method. Cells report mean log response ratio (LRR) for yield; shading encodes effect direction and magnitude, and grey cells indicate no data. Asterisks denote significance (*p < 0.05, ***p < 0.001).

The species-by-extraction analysis highlighted additional heterogeneity in response magnitude (**Figure 7**). Water-based and alkaline extraction methods were associated with positive responses across multiple taxa, with most combinations clustering below 25%, while a limited number of species–extraction combinations exhibited higher point estimates exceeding 30% and, in isolated cases >60%, accompanied by wide confidence intervals. Other combinations showed low or non-significant responses.

## 4 Discussion

Seaweed extract–based biostimulants increased crop yield and biomass by approximately 16% across studies worldwide, indicating a broadly positive agronomic effect across diverse production environments. These consistent benefits across climates, stress conditions, and trial types suggest that seaweed extracts enhance key physiological processes such as nutrient uptake, resource use efficiency, and plant stress tolerance. Stronger responses observed in tropical and arid regions likely reflect the higher prevalence of abiotic stresses in these environments, where improvements in physiological resilience can translate more directly into yield gains. Indeed, the combination of drought and heat stress is known to cause severe yield reductions, particularly in tropical and subtropical regions (Sánchez-Bermúdez et al., 2022; Alghamdi, 2024; Tran et al., 2025). Accordingly, a meta-analysis conducted by Li et al. (2022) reported average yield gains of 17.9% in open-field systems following the application of different types of biostimulants, with the greatest benefits occurring under arid climates. However, evidence from cooler climates remains limited, highlighting the need for additional studies to better understand the consistency of these responses across environmental contexts.

Yield increases of 12–18% were observed across contrasting soil types, largely independent of phosphorus, potassium, pH, salinity, or organic matter levels. This contrasts with the global meta-analysis by Li et al. (2022), which reported higher biostimulant efficacy under low organic matter, non-neutral pH, salinity, nutrient deficiencies, and sandy textures. When restricting the analysis to seaweed-based biostimulants (198 studies), however, a distinct response pattern emerged. Consistent with previous reports, seaweed extracts enhance root development, resource use efficiency, antioxidant activity, chlorophyll content, and nutrient accumulation across a wide range of soil conditions (Pereira et al., 2020; Kumari et al., 2023; Pienaar et al., 2025). Relative yield gains were greater in slightly to moderately saline soils and finer-textured soils, suggesting that soil properties modulate, but do not constrain, the benefits of seaweed biostimulants. Under salinity, osmoprotectants contribute to osmotic adjustment, ion homeostasis, and stress tolerance, while regulation of ROS-scavenging pathways and membrane stabilization enhances resilience and yield (Kumar et al., 2020; Pereira et al., 2020; Deolu-Ajayi et al., 2022; Trivedi et al., 2023). In finer-textured soils, seaweed-derived polysaccharides may further improve nutrient retention and root–soil interactions (Yasmeen et al., 2025). Collectively, these mechanisms may explain why relative yield gains under moderate edaphic stress can exceed 25%. Interpretation of soil moderators, however, remains constrained by methodological heterogeneity, including phosphorus and potassium extraction methods (Horneck et al., 2011; Hazelton and Murphy, 2016), variability in electrical conductivity measurements (Kargas et al., 2018), and the dynamic nature of pH and salinity indicators (Smith and Doran, 1997), underscoring the need for standardized classification and normalization in soil meta-analyses.

The overall positive yield response confirms the effectiveness of seaweed extract-based biostimulant treatment, although the magnitude of its impact varied among crop groups. Vegetables and legumes showed the strongest responses, likely reflecting greater physiological sensitivity and nutrient demand, consistent with previous meta-analyses (Li et al., 2022). Cereals, fruit trees, nuts, and sugar crops exhibited more moderate gains, possibly due to differences in growth strategies, nutrient uptake, or inherent physiology. In contrast, roots and tubers showed no significant effects, suggesting that yield determinants such as storage organ development may be less influenced by the mechanisms associated with seaweed extract application. Oilseed crops showed a relatively high estimated response, but the limited dataset warrants cautious interpretation (Szparaga et al., 2018). Together, these findings highlight the need for crop-specific application strategies that account for differences in crop physiology and production systems.

These crop-group differences also indicate that the practical relevance of seaweed extract application cannot be interpreted from yield response alone. Previous comparative syntheses have shown that crop performance varies substantially across categories and production contexts (de Ponti et al., 2012; Seufert et al., 2012). This pattern became even more apparent when crop-group responses were interpreted together with weighted producer prices, as groups with more moderate biological responses but higher crop value may still have substantial economic relevance. This interpretation should, however, be viewed as a complementary gross-value context rather than a formal profitability assessment, because broader financial outcomes also depend on input costs, labor requirements, and market conditions not captured in the present synthesis (Crowder and Reganold, 2015).

Extract effectiveness appears to depend more on timing, frequency, and environment than on fixed dose. The lack of a clear dose–response relationship indicates that plants benefit within a broad concentration range, with early-stage applications (BBCH 1–3) most consistently enhancing root establishment, nutrient uptake, and stress resilience. Moderate application frequencies (4–6 applications) stimulate growth without negative effects, whereas very low or high frequencies can produce inconsistent results (Andreotti et al., 2022). The higher yield response observed for soil-directed applications of seaweed extracts (fertigation and drenching) likely results from their direct delivery to the rhizosphere, stimulating root growth, enhancing nutrient uptake (Khan et al., 2009; Pereira et al., 2020; Kumari et al., 2023; Pienaar et al., 2025), and promoting beneficial microbial activity (Shukla et al., 2019). In contrast, foliar applications primarily influence aboveground tissues by improving photosynthesis, delaying senescence, and enhancing stress tolerance (Pereira et al., 2020; Kumari et al., 2023; Pienaar et al., 2025), while seed treatments mainly act during germination and early seedling development (Ribeiro et al., 2024), generally resulting in more transient effects.

These agronomic patterns are consistent with plausible biological mechanisms underlying seaweed extract activity. Alkaline extraction can disrupt algal cell walls and enhance the release of bioactive compounds such as polysaccharides and phenolics that influence plant signaling and stress responses (Craigie, 2011; Zhang et al., 2024). The higher effectiveness of soil-directed applications likely reflects the delivery of these compounds to the rhizosphere, where they may stimulate root development, improve nutrient uptake, and interact with beneficial microbial communities (Khan et al., 2009; Shukla et al., 2019; Alahmad et al., 2023). Early-stage applications coincide with phases of rapid root establishment and high developmental plasticity, enabling stronger responses through improved nutrient acquisition and physiological priming (Wally et al., 2013; Andreotti et al., 2022). Together, these mechanisms provide a biologically plausible explanation for the moderator effects identified in this meta-analysis.

Seaweed biostimulants are widely used in agriculture and show positive effects on crop growth, but the specific bioactive compounds and mechanisms of action remain incompletely understood (Pereira et al., 2020; Kumari et al., 2023; Pienaar et al., 2025). While additional field trials are needed to refine optimal concentrations and assess crop-specific responses and economic viability, current evidence suggests that dose alone is not the primary driver of plant response. Consequently, future research should focus on other critical variables such as seaweed species, extraction method, formulation type, and application timing. Additional benefits may also arise when seaweed extracts are combined with other bioinputs rather than used individually. *Bacillus*- and *Trichoderma*-based biofertilizers, for example, can improve soil health and crop productivity by stimulating beneficial microbial communities, with *Bacillus* promoting bacterial groups and Trichoderma favoring fungi involved in soil pH regulation (Zhang et al., 2025). Because seaweed extracts can act as prebiotic substrates, their integration with beneficial microorganisms or other bioinputs may represent a synergistic strategy to enhance soil fertility, maintain microbial diversity, and improve crop performance (Alahmad et al., 2023).

The effectiveness of algae-based products also depends on formulation, extraction method, and algal species. Powdered formulations generally outperformed liquid ones, likely due to their greater stability and higher concentration of active compounds, as reported by Espinosa-Antón et al. (2023), who observed stronger morphological responses in tomato plants treated with powdered *Ulva ohnoi*. Similarly, alkaline hydrolysis appears to be particularly effective in releasing bioactive molecules from algal cell walls (Zhang et al., 2024). Among macroalgae, *Ascophyllum nodosum* consistently provides the most reliable yield increases and closely aligns with the average reported effect of 16–17%, which may explain why this species dominates the biostimulant market (Khan et al., 2009). Its effectiveness is often attributed to metabolites such as phenolics, mannitol, laminarin, and sulfated fucoidans that evolved as adaptations to the harsh intertidal environments where the species occurs (Ugarte et al., 2006; Rayorath et al., 2008; Shukla et al., 2019; Yakhin et al., 2017). Other species, including *Laminaria*, *Gracilaria*, and *Kappaphycus*, may sometimes produce stronger but more variable agronomic responses (Khan et al., 2009). Considering the vast biodiversity of marine macroalgae and the relatively recent expansion of research in this field (Yakhin et al., 2017; Prisa et al., 2024), it is likely that additional species with promising agricultural potential will be identified in the future.

## 5 Conclusions

This meta-analysis shows that seaweed extract–based biostimulants increase crop yield by approximately 16% across diverse agronomic conditions, indicating consistent benefits despite substantial variability among studies. Stronger responses were observed in tropical and arid climates, while soil properties generally acted as modulators rather than primary drivers of crop response. Yield gains also varied among crop groups, with vegetables, legumes, and cereals showing the most pronounced improvements. The magnitude and consistency of responses were influenced by application strategy and product characteristics, with early phenological applications, repeated treatments, soil-directed delivery, and powder formulations or alkaline/water-based extracts generally producing stronger effects; among source species, *Ascophyllum nodosum* provided the most consistent performance. Overall, these findings highlight the potential of seaweed extract–based biostimulants as a promising tool to enhance crop productivity and resilience under variable environmental conditions, although their effectiveness depends on optimized application strategies and formulation properties. Despite the large dataset analyzed, the interpretation of subgroup differences should consider the substantial between-study heterogeneity and the uneven representation of certain categories, which may influence the precision of some estimates. Further research should therefore focus on clarifying the underlying physiological and microbiological mechanisms and refining agronomic recommendations to optimize the use of seaweed-based biostimulants across different cropping systems.

## Data Availability

The datasets presented in this study can be found in online repositories. The names of the repository/repositories and accession number(s) can be found in the article/**Supplementary Material**.
