# Supplementary material for: Crop yield responses to seaweed extract-based biostimulants depend on application strategy, formulation, and extraction methods: a meta-analysis
Source: Front Plant Sci. 2026 Apr 21;17:1803269. doi: 10.3389/fpls.2026.1803269 (PMC13139174; doi:10.3389/fpls.2026.1803269)
Supplement: Supplementary file 1 [file Supplementaryfile1.docx]

Supplementary Material

# Supplementary Data

## Overall Effect

Model:
Effect size (LRR): 0.1468 [0.1249, 0.1687]
Percent change: 15.8% [13.3%, 18.4%]
P-value: 0.0000*
Number of studies included: 192
Total observations: 2021
Studies excluded (n=1): 6
I²: 92.8% (heterogeneity)
τ²: 0.0153 (between-study variance)
Q-test: 2765.54 (p = 0.0000)

## Egger’s Test

Egger's regression test indicated a significant asymmetry in the funnel plot (bias coefficient = 0.40, p = 0.019), suggesting the possible presence of publication bias.

After performing the publication bias analysis on the global meta-analysis, the following results were observed:

- Original effect: an increase of 15.8% (LRR = 0.147; 95% CI: 13.3–18.4%), highly significant (p < 0.001).
- Trim-and-Fill: the method identified the possible absence of 33 studies on the left side of the funnel plot, which reduced the estimated effect to +11.0% (95% CI: 8.2–13.8%), remaining significant.

Although the results suggest the possible presence of publication bias, the effect remains positive, robust, and statistically significant in both scenarios. The magnitude of the effect varies between +11% and +16%, depending on the adjustment, but in both cases indicates a consistent improvement.

# Supplementary Figures and Tables


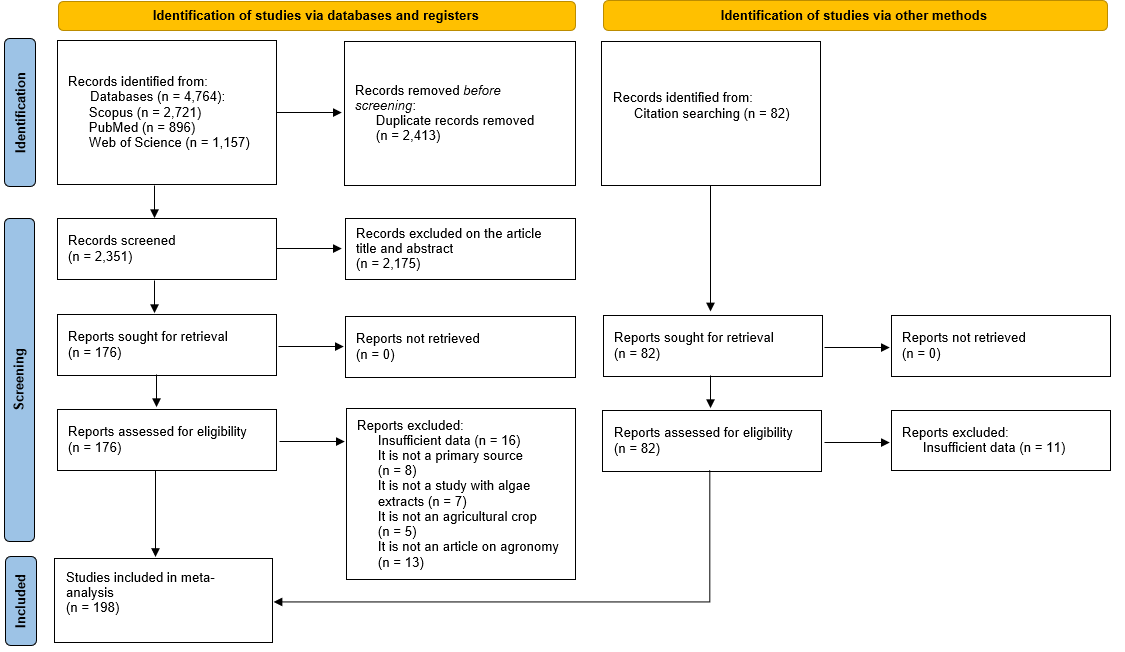


**Supplementary Figure 1.** PRISMA 2020 flow diagram of study selection. Sources: Scopus, PubMed, Web of Science and citation searching; duplicates removed before screening. Boxes show counts (n) and reasons for exclusion. 198 studies were included.


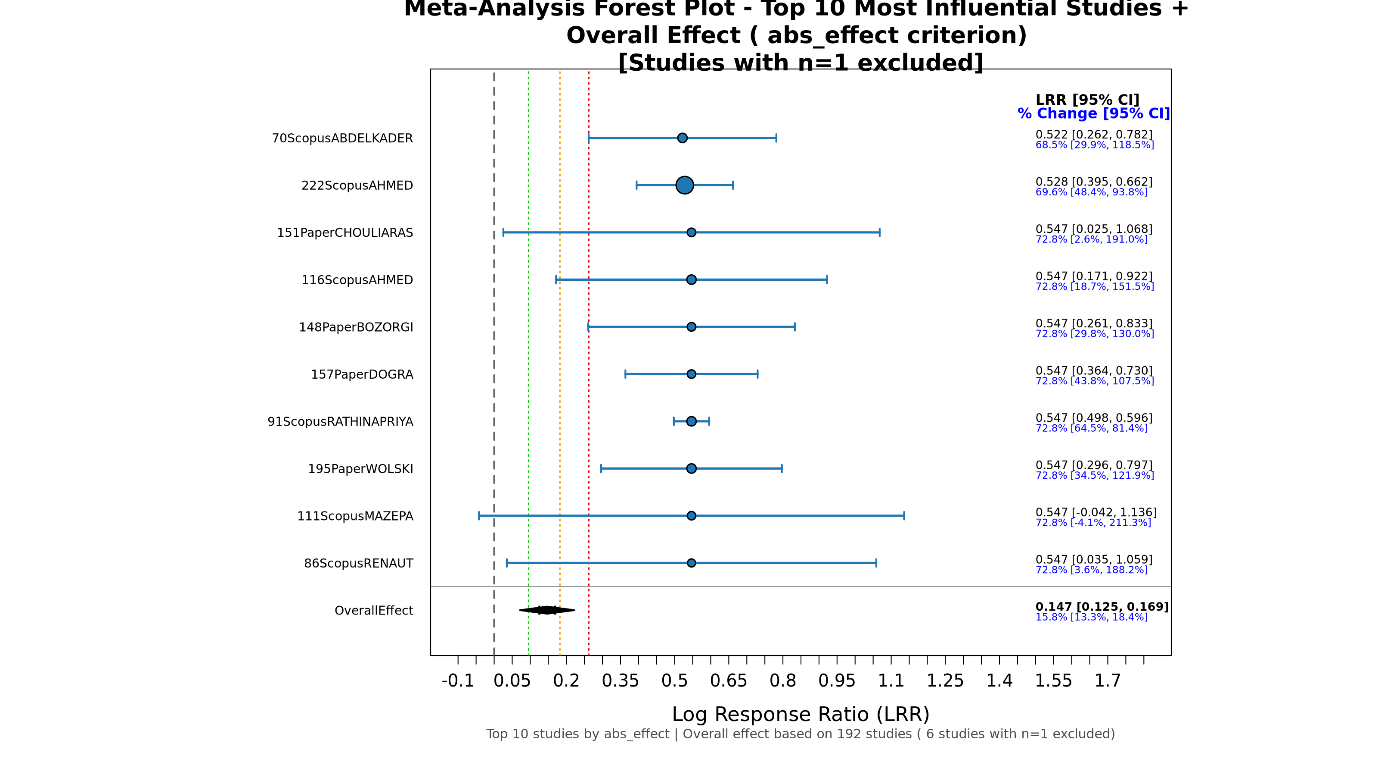


**Supplementary Figure 2.** Forest plot – Top 10 most influential studies and overall effect (absolute effect criterion). Studies with n = 1 were excluded.


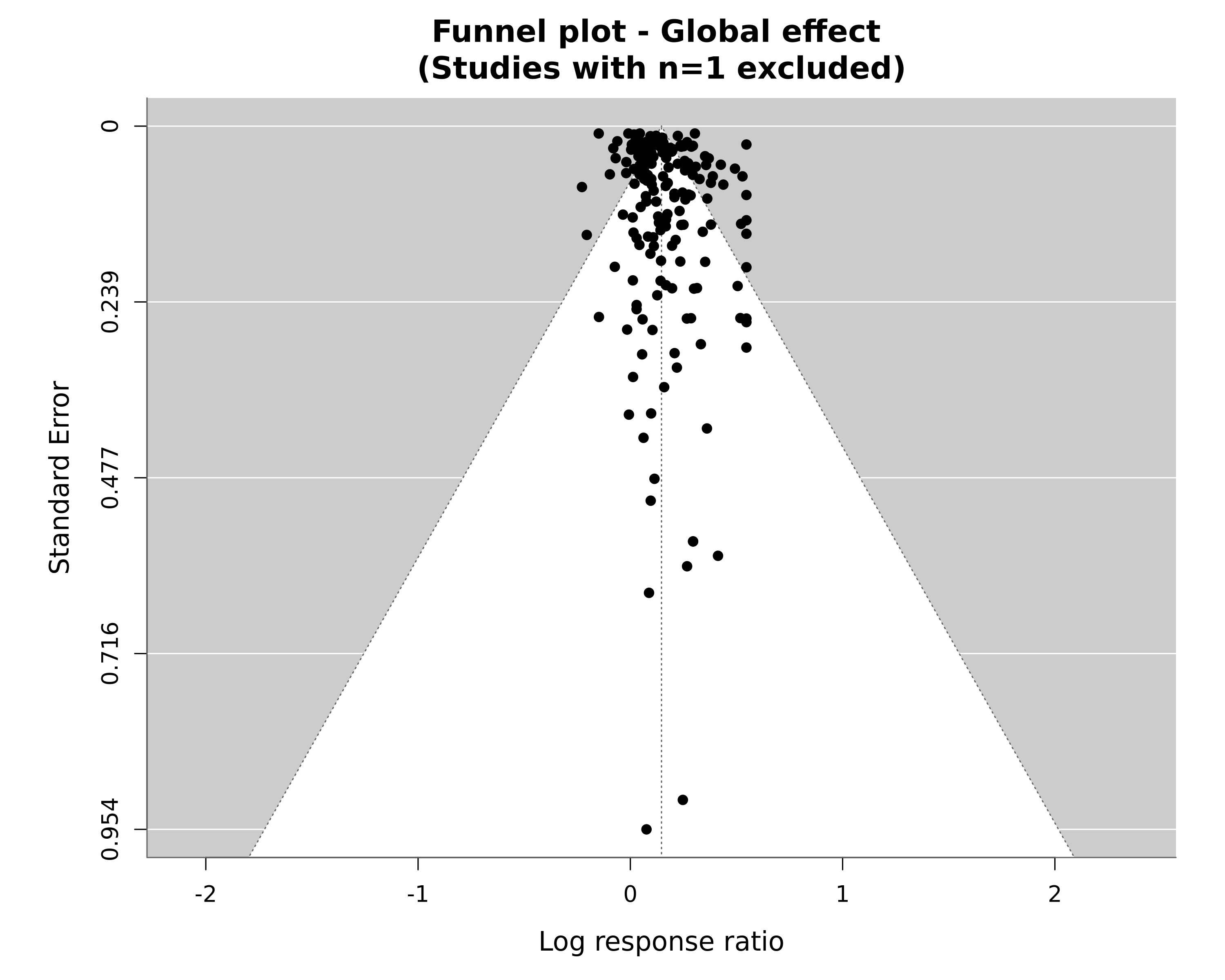


**Supplementary Figure 3.** Funnel plot – Global effect (studies with n = 1 excluded).


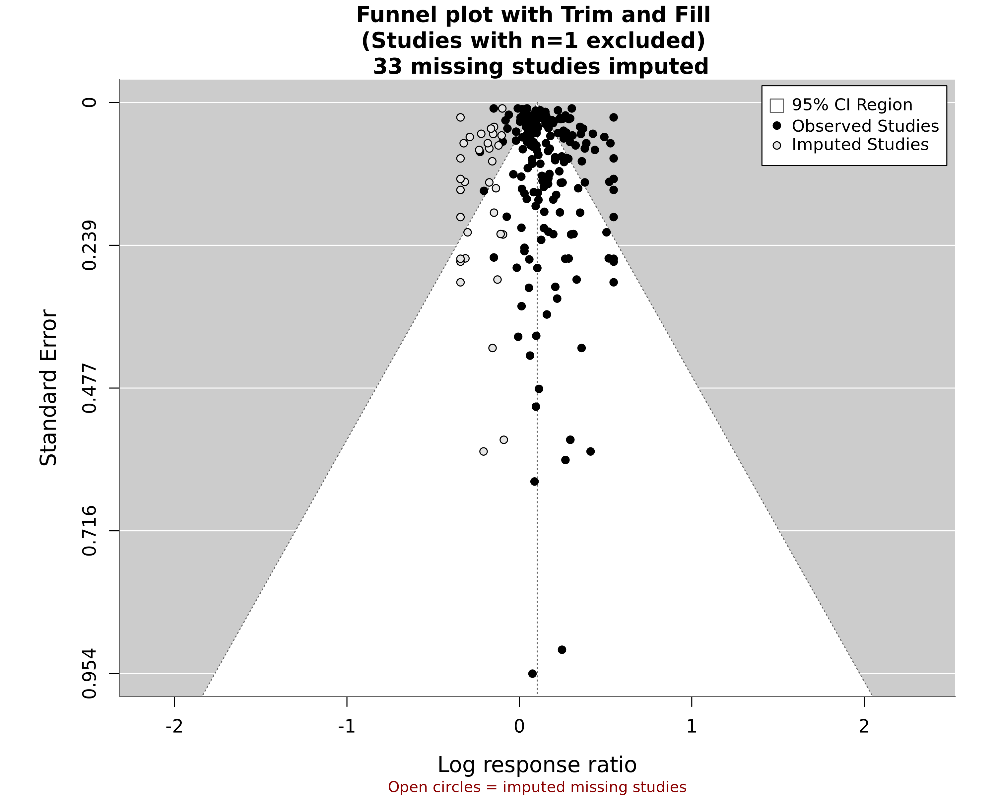


**Supplementary Figure 3.** Funnel plot with Trim-and-Fill (studies with n = 1 excluded), showing 33 missing studies imputed.


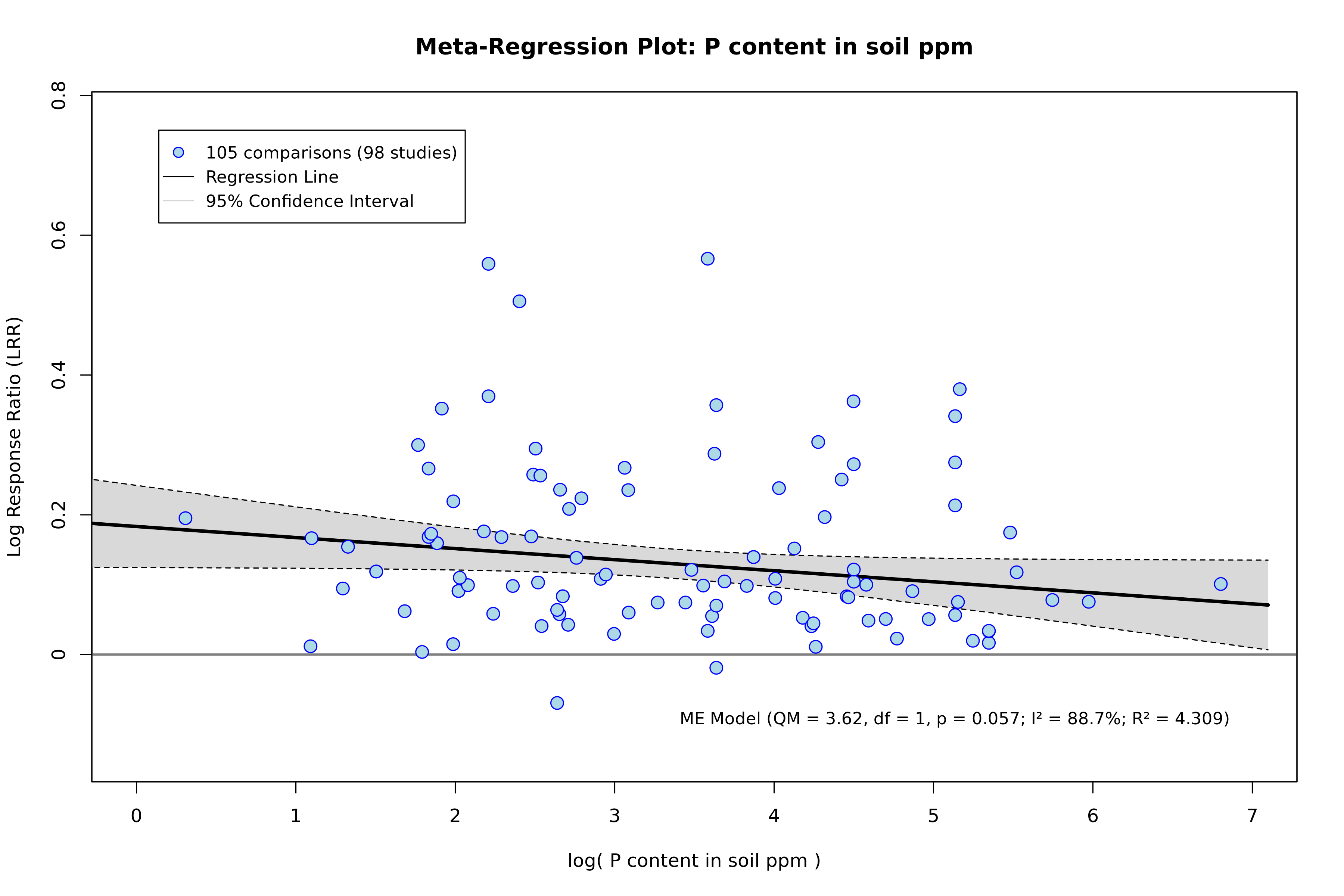


**Supplementary Figure 4.** Meta-regression of crop yield response against soil phosphorus content. Meta-regression analysis evaluating the relationship between soil phosphorus content and crop yield response expressed as log response ratio (LRR). Points represent individual comparisons, with point size proportional to study weight. The solid line indicates the fitted mixed-effects meta-regression model, and the shaded area represents the 95% confidence interval. The model indicates a weak negative trend, with confidence intervals overlapping zero.


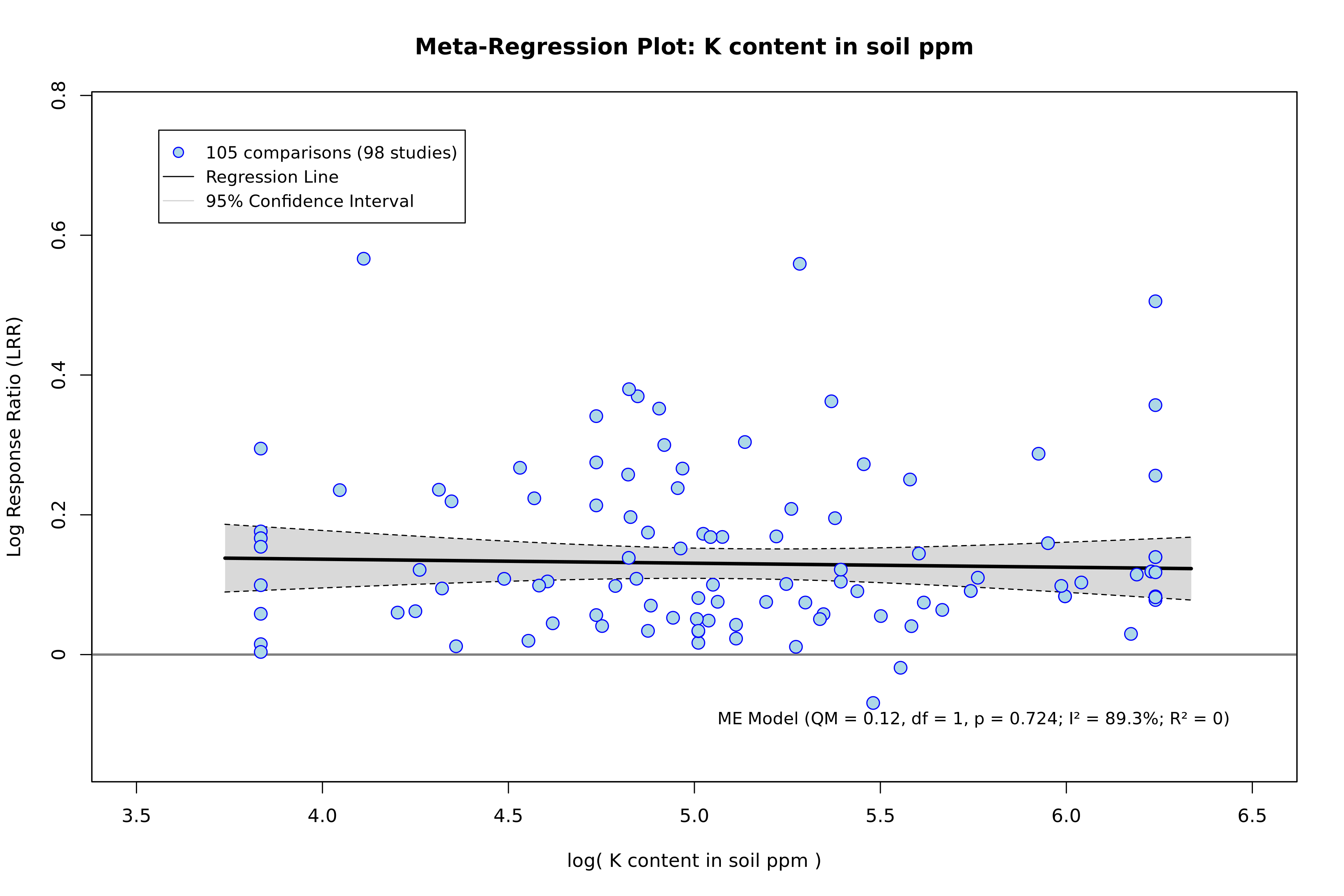


**Supplementary Figure 5.** Meta-regression of crop yield response against soil potassium content.
Meta-regression analysis evaluating the relationship between soil potassium content and crop yield response expressed as log response ratio (LRR). Points represent individual comparisons, with point size proportional to study weight. The solid line indicates the fitted mixed-effects meta-regression model, and the shaded area represents the 95% confidence interval. The model shows no consistent linear relationship between soil potassium content and yield response.


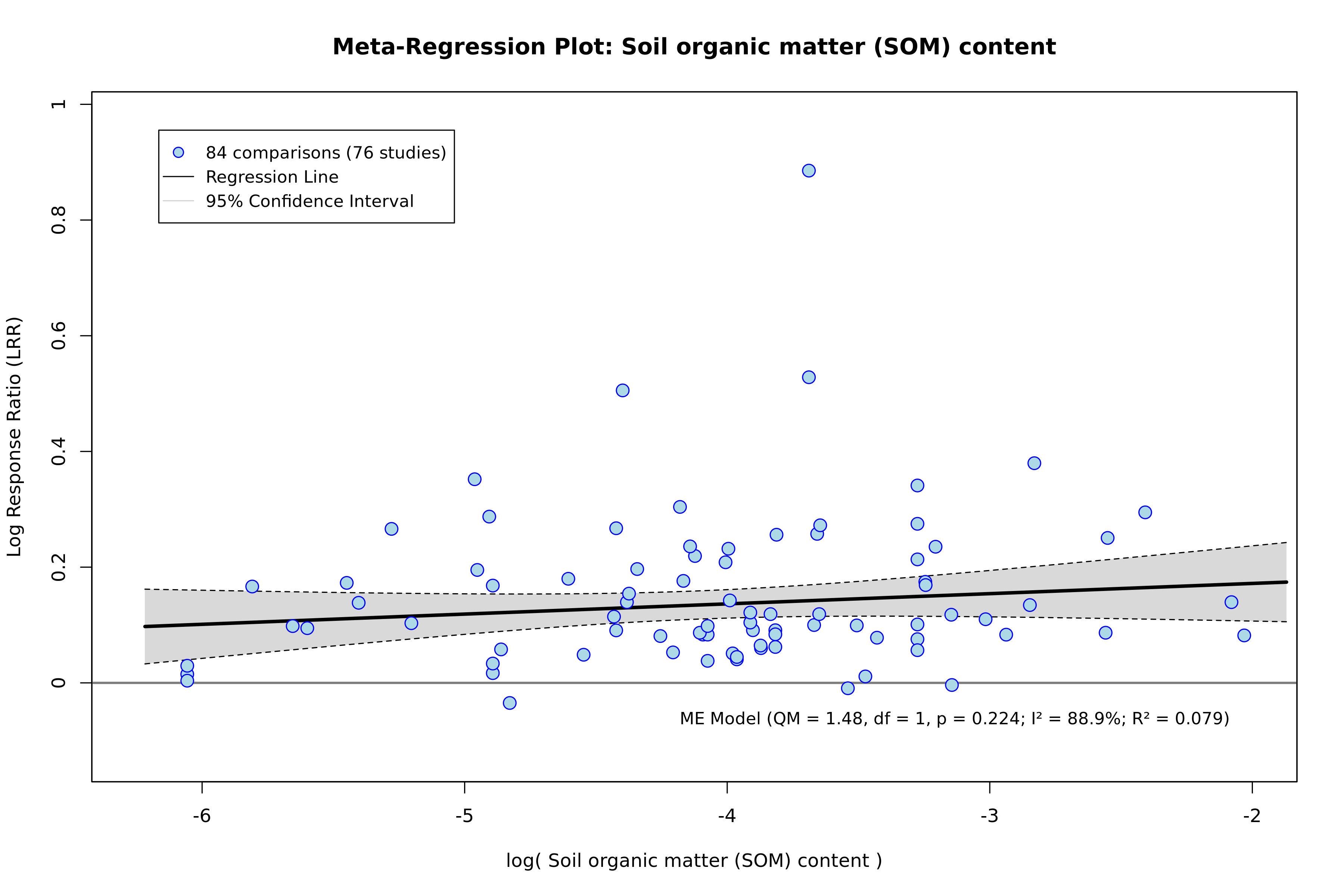


**Supplementary Figure 6.** Meta-regression of crop yield response against soil organic matter content. Meta-regression analysis evaluating the relationship between soil organic matter content and crop yield response expressed as log response ratio (LRR). Points represent individual comparisons, with point size proportional to study weight. The solid line indicates the fitted mixed-effects meta-regression model, and the shaded area represents the 95% confidence interval. The model shows a weak and non-significant linear relationship between soil organic matter content and yield response.


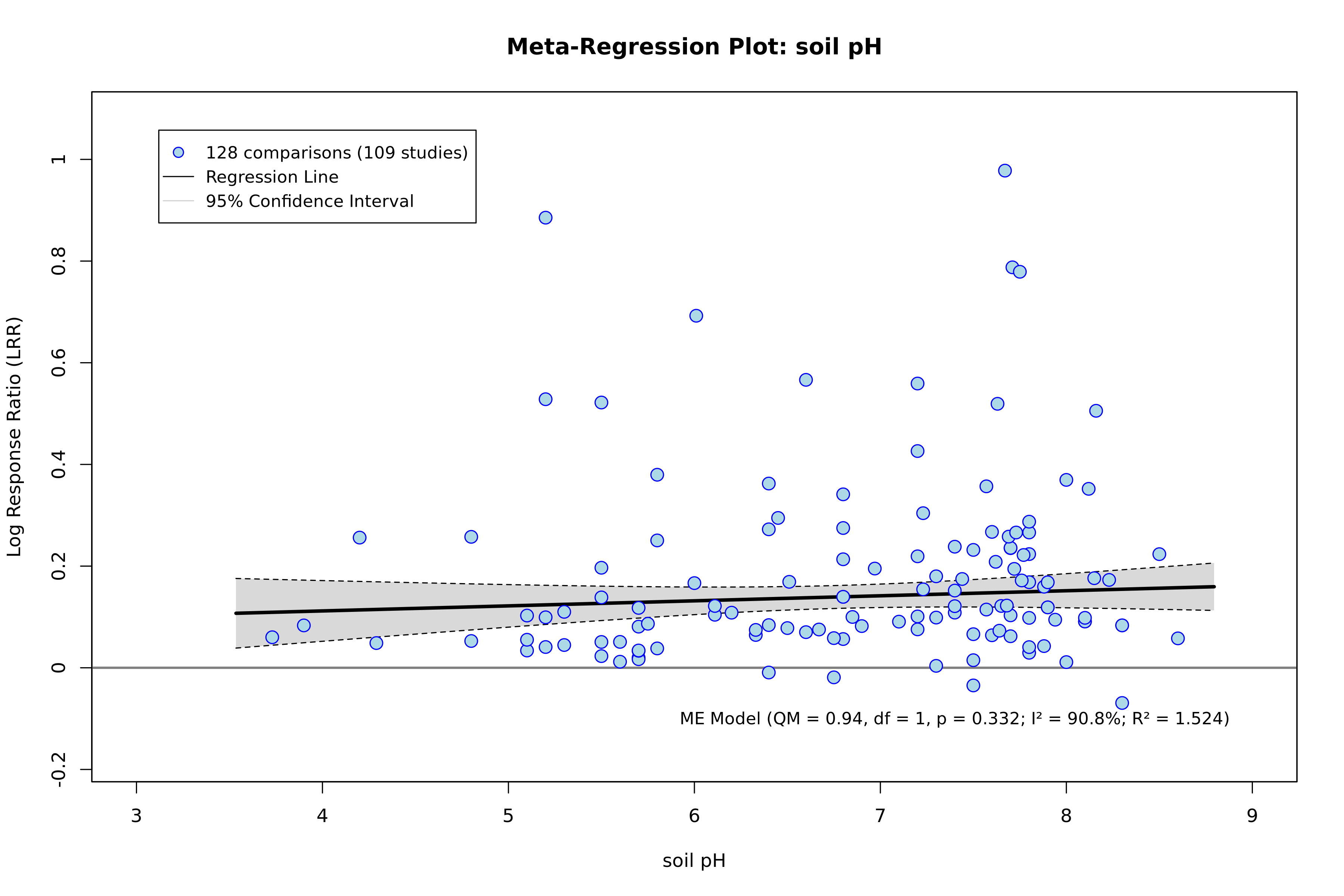


**Supplementary Figure 7.** Meta-regression of crop yield response against soil pH. Meta-regression analysis evaluating the relationship between soil pH and crop yield response expressed as log response ratio (LRR). Points represent individual comparisons, with point size proportional to study weight. The solid line indicates the fitted mixed-effects meta-regression model, and the shaded area represents the 95% confidence interval. The model shows a weak and non-significant linear relationship between soil pH and yield response.


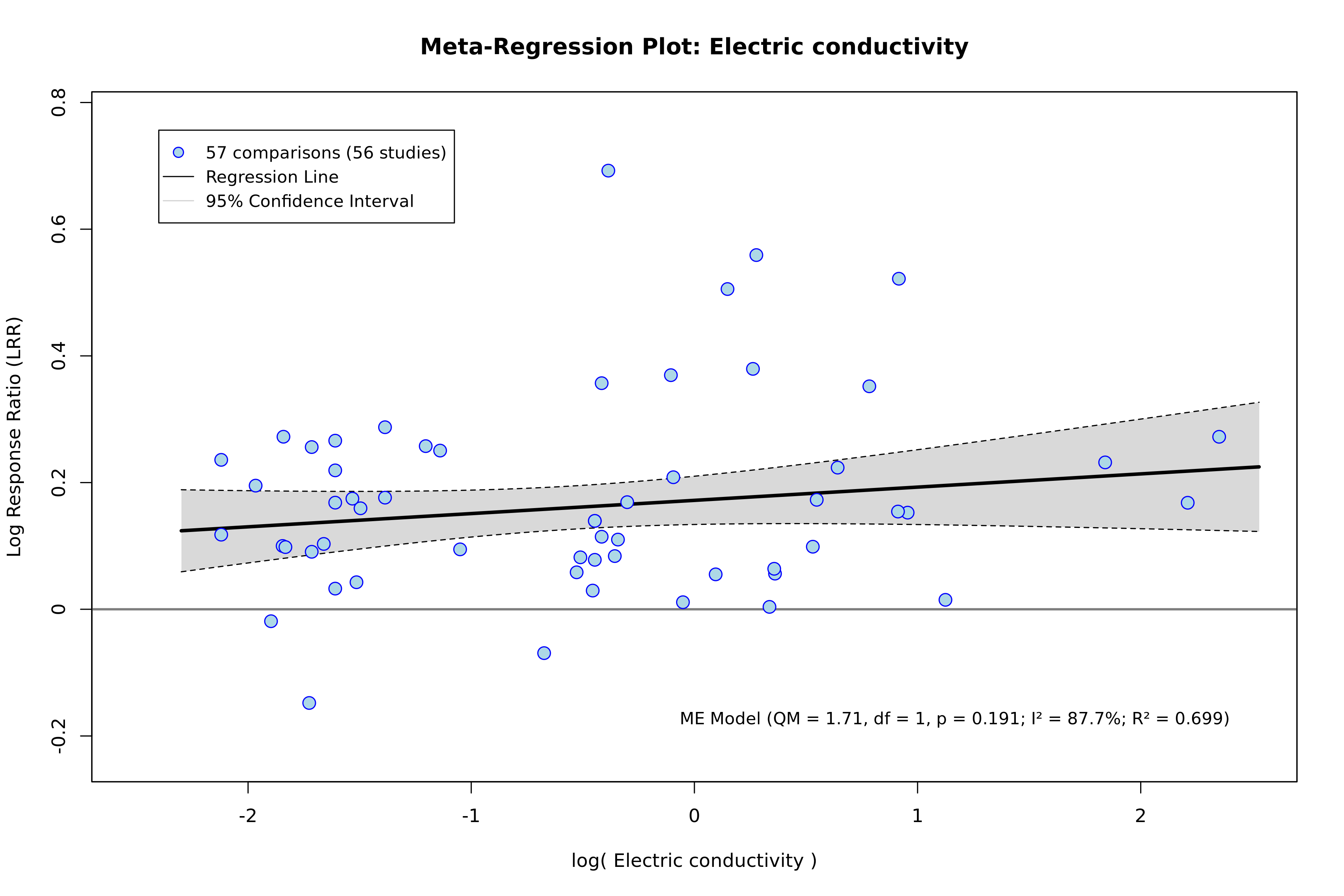


**Supplementary Figure 8.** Meta-regression of crop yield response against soil electrical conductivity.
Meta-regression analysis evaluating the relationship between soil electrical conductivity and crop yield response expressed as log response ratio (LRR). Points represent individual comparisons, with point size proportional to study weight. The solid line indicates the fitted mixed-effects meta-regression model, and the shaded area represents the 95% confidence interval. The model shows a weak and non-significant linear relationship between soil electrical conductivity and yield response.


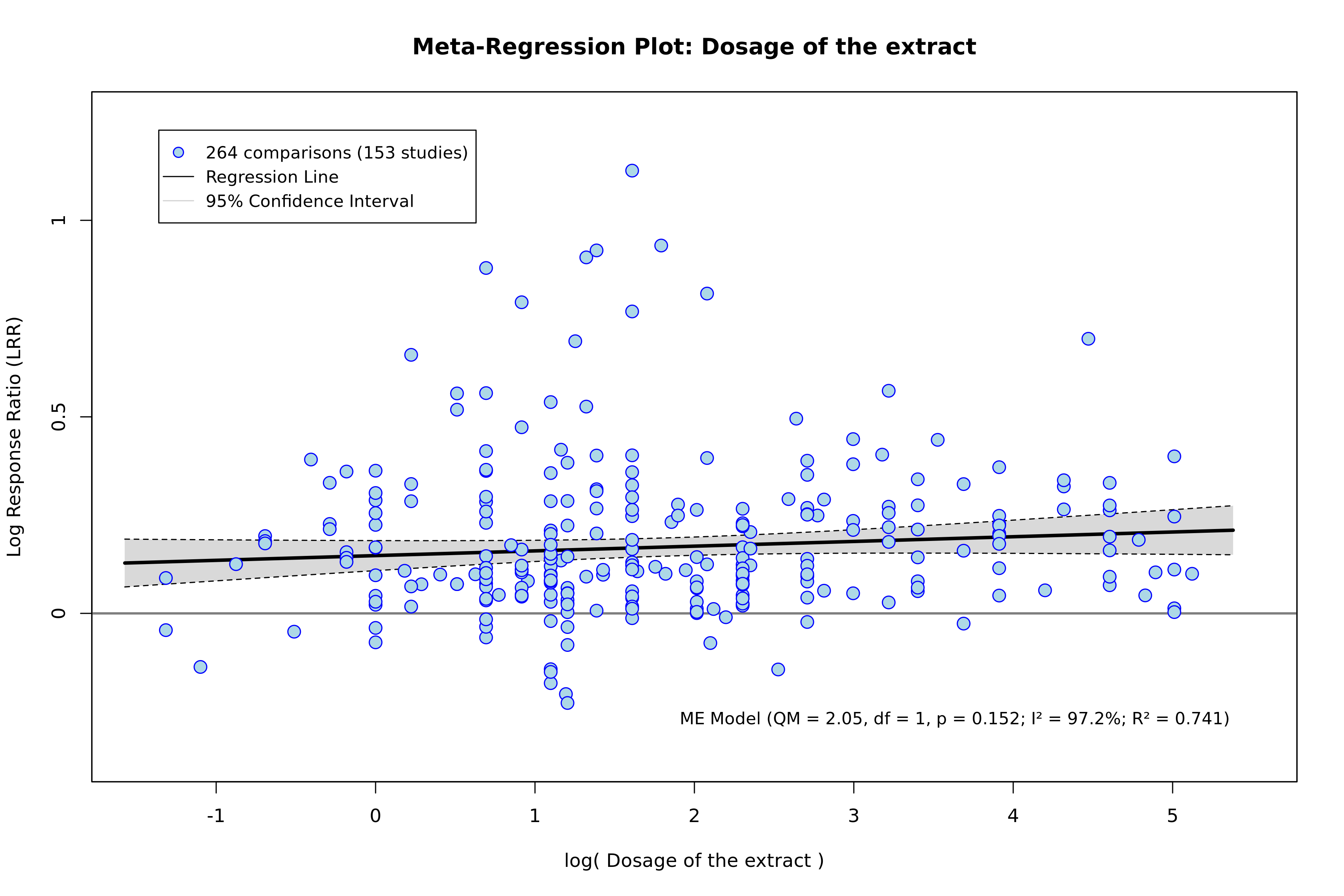


**Supplementary Figure 9.** Meta-regression of crop yield response against extract dosage.
Meta-regression analysis evaluating the relationship between extract dosage and crop yield response expressed as log response ratio (LRR). Points represent individual comparisons, with point size proportional to study weight. The solid line indicates the fitted mixed-effects meta-regression model, and the shaded area represents the 95% confidence interval. The model indicates no clear linear dose–response relationship across the range of extract dosages evaluated.

**References**

Egger, M., Smith, G. D., Schneider, M., and Minder, C. (1997). Bias in meta-analysis detected by a simple, graphical test. *BMJ* 315, 629–634. https://doi.org/10.1136/bmj.315.7109.629
