# Supplementary material for: Crop yield responses to seaweed extract-based biostimulants depend on application strategy, formulation, and extraction methods: a meta-analysis
Source: Front Plant Sci. 2026 Apr 21;17:1803269. doi: 10.3389/fpls.2026.1803269 (PMC13139174; doi:10.3389/fpls.2026.1803269)
Supplement: Supplementary file 4 [file Supplementaryfile4.docx]

**Supplementary Material S4. List of papers used in the meta-analysis**

1. Abbas, M., Anwar, J., Zafar-Ul-Hye, M., Khan, R. I., Saleem, M., Rahi, A. A., Danish, S., & Datta, R. (2020). Effect of seaweed extract on productivity and quality attributes of four onion cultivars. *Horticulturae*, *6*(2). https://doi.org/10.3390/horticulturae6020028
2. Abdelkader, M. M., Gaplaev, M. S., Terekbaev, A. A., & Puchkov, M. Y. (2021). The Influence of Biostimulants on Tomato Plants Cultivated under Hydroponic Systems. *Journal of Horticultural Research*, *29*(2), 107–116. https://doi.org/10.2478/johr-2021-0012
3. Ahmed, M., Ullah, H., Attia, A., Tisarum, R., Cha-um, S., & Datta, A. (2023). Interactive Effects of Ascophyllum nodosum Seaweed Extract and Silicon on Growth, Fruit Yield and Quality, and Water Productivity of Tomato under Water Stress. *Silicon*, *15*(5), 2263–2278. https://doi.org/10.1007/s12633-022-02180-x
4. Ahmed, M., Ullah, H., Himanshu, S. K., García-Caparrós, P., Tisarum, R., Cha-um, S., & Datta, A. (2024). Ascophyllum nodosum seaweed extract and potassium alleviate drought damage in tomato by improving plant water relations, photosynthetic performance, and stomatal function. *Journal of Applied Phycology*, *36*(4), 2255–2268. https://doi.org/10.1007/s10811-024-03266-2
5. Alam, M. Z., Braun, G., Norrie, J., & Hodges, D. M. (2014). Ascophyllum extract application can promote plant growth and root yield in carrot associated with increased root-zone soil microbial activity. *Canadian Journal of Plant Science*, *94*(2), 337–348. https://doi.org/10.4141/CJPS2013-135
6. Al-Bayati, A. S., Sadaq Jaafar, H., Jubair, N., & Alhasnawi, R. (2020). Evaluation of eggplant via different drip irrigation intervals and foliar sprays with seaweed extract biostimulant evaluation of eggplant via different drip irrigation intervals and foliar sprays with seaweed extract biostimulant evaluation of eggplant via different drip irrigation intervals and foliar sprays with seaweed extract biostimulant. In *Article in International Journal of Agricultural and Statistical Sciences*. https://www.researchgate.net/publication/347937561
7. Ali, M. (2024). Yield and physio-biochemical properties of wheat grain beneath organic and inorganic fertilization and application of biostimulator. https://doi.org/10.15666/aeer
8. Ali, N., Farrell, A., Ramsubhag, A., & Jayaraman, J. (2016). The effect of Ascophyllum nodosum extract on the growth, yield and fruit quality of tomato grown under tropical conditions. *Journal of Applied Phycology*, *28*(2), 1353–1362. https://doi.org/10.1007/s10811-015-0608-3
9. Ali, O., Farrell, A. D., Ramsubhag, A., & Jayaraman, J. (2024a). Beneficial effects of an Ascophyllum nodosum extract on tomato (Solanum lycopersicum L.) during water stress. *Journal of Applied Phycology*, *36*(1), 385–397. https://doi.org/10.1007/s10811-023-03156-z
10. Ali, O., Farrell, A. D., Ramsubhag, A., & Jayaraman, J. (2024b). Beneficial effects of an Ascophyllum nodosum extract on tomato (Solanum lycopersicum L.) during water stress. *Journal of Applied Phycology*, *36*(1), 385–397. https://doi.org/10.1007/s10811-023-03156-z
11. Ali, O., Ramsubhag, A., & Jayaraman, J. (2022). Transcriptome-wide modulation by Sargassum vulgare and Acanthophora spicifera extracts results in a prime-triggered plant signalling cascade in tomato and sweet pepper. *AoB PLANTS*, *14*(6). https://doi.org/10.1093/aobpla/plac046
12. Ali, O., Ramsubhag, A., & Jayaraman, J. (2023). Application of extracts from Caribbean seaweeds improves plant growth and yields and increases disease resistance in tomato and sweet pepper plants. *Phytoparasitica*, *51*(4), 727–745. https://doi.org/10.1007/s12600-022-01035-w
13. Amato, G., Cardone, L., Cicco, N., Denora, M., Perniola, M., Casiello, D., de Martino, L., de Feo, V., & Candido, V. (2024). Morphological traits, yield, antioxidant activity and essential oil composition of oregano as affected by biostimulant foliar applications. *Industrial Crops and Products*, *222*. https://doi.org/10.1016/j.indcrop.2024.119702
14. Amer, H. M., Marrez, D. A., Salama, A. B., Wahba, H. E., & Khalid, K. A. (2019). Growth and chemical constituents of cardoon plant in response to foliar application of various algal extracts. *Biocatalysis and Agricultural Biotechnology*, *21*. https://doi.org/10.1016/j.bcab.2019.101336
15. Aremu, A. O., Makhaye, G., Tesfay, S. Z., Gerrano, A. S., du Plooy, C. P., & Amoo, S. O. (2022). Influence of Commercial Seaweed Extract and Microbial Biostimulant on Growth, Yield, Phytochemical Content, and Nutritional Quality of Five Abelmoschus esculentus Genotypes. *Agronomy*, *12*(2). https://doi.org/10.3390/agronomy12020428
16. Arioli, T., Mattner, S. W., Hepworth, G., Mcclintock, D., & Mcclinock, R. (n.d.). *Effect of seaweed extract application on wine grape yield in Australia*. https://doi.org/10.1007/s10811-021-02423-1/Published
17. Arioli, T., Villalta, O. N., Hepworth, G., Farnsworth, B., & Mattner, S. W. (2024a). Effect of seaweed extract on avocado root growth, yield and post-harvest quality in far north Queensland, Australia. *Journal of Applied Phycology*, *36*(2), 745–755. https://doi.org/10.1007/s10811-023-02933-0
18. Arioli, T., Villalta, O. N., Hepworth, G., Farnsworth, B., & Mattner, S. W. (2024b). Effect of seaweed extract on avocado root growth, yield and post-harvest quality in far north Queensland, Australia. *Journal of Applied Phycology*, *36*(2), 745–755. https://doi.org/10.1007/s10811-023-02933-0
19. Author, C., Yunsheng, L., Zhu, O., & Sawan, O. M. (2012). ORIGINAL ARTICLES Response of Garlic (Allium Sativum, L.) Plants To Foliar Spraying of Some Bio-Stimulants Under Sandy Soil Condition. In *Journal of Applied Sciences Research* (Vol. 8, Issue 2).
20. Baghdadi, A., della Lucia, M. C., Borella, M., Bertoldo, G., Ravi, S., Zegada-Lizarazu, W., Chiodi, C., Pagani, E., Hermans, C., Stevanato, P., Nardi, S., Monti, A., & Mangione, F. (2022). A dual-omics approach for profiling plant responses to biostimulant applications under controlled and field conditions. *Frontiers in Plant Science*, *13*. https://doi.org/10.3389/fpls.2022.983772
21. Bozorgi, H. R. (2012). *ARPN Journal of Agricultural and Biological Science* effects of foliar spraying with marine plant ascophyllum nodosum extract and nano iron chelate fertilizer on fruit yield and several attributes of eggplant *(Solanum melongena L.)*. *7*(5). www.arpnjournals.com
22. Brahmachari, K., Chandra, B., Viswavidyalaya, K., Pramanick, B., Ghosh, A., & Zodape, & S. T. (2015). Effect of seaweed saps derived from two marine algae Kappaphycus and Gracilaria on growth and yield improvement of blackgram. In *Article in Indian Journal of Geo-Marine Sciences* (Vol. 45, Issue 6). https://www.researchgate.net/publication/301899099
23. Castellano-Hinojosa, A., Meyering, B., Nuzzo, A., Strauss, S. L., & Albrecht, U. (2021). Effect of plant biostimulants on root and plant health and the rhizosphere microbiome of citrus trees in huanglongbing-endemic conditions. *Trees - Structure and Function*, *35*(5), 1525–1539. https://doi.org/10.1007/s00468-021-02133-8
24. Chanthini, K. M. P., Stanley-Raja, V., Thanigaivel, A., Karthi, S., Palanikani, R., Sundar, N. S., Sivanesh, H., Soranam, R., & Senthil-Nathan, S. (2019). Sustainable agronomic strategies for enhancing the yield and nutritional quality ofwild tomato, solanum lycopersicum (1) Var Cerasiforme Mill. *Agronomy*, *9*(6). https://doi.org/10.3390/agronomy9060311
25. Chaski, C., Giannoulis, K. D., Alexopoulos, A. A., & Petropoulos, S. A. (2023). Biostimulant Application Alleviates the Negative Effects of Deficit Irrigation and Improves Growth Performance, Essential Oil Yield and Water-Use Efficiency of Mint Crop. *Agronomy*, *13*(8). https://doi.org/10.3390/agronomy13082182
26. Chaudhary, D. (2011). Foliar application of seaweed sap as biostimulant for enhancement of yield and quality of tomato (Lycopersicon esculentum Mill.). In *Article in Journal of Scientific & Industrial Research*. https://www.researchgate.net/publication/235545368
27. Chen, D., Zhou, W., Yang, J., Ao, J., Huang, Y., Shen, D., Jiang, Y., Huang, Z., & Shen, H. (2021). Effects of Seaweed Extracts on the Growth, Physiological Activity, Cane Yield and Sucrose Content of Sugarcane in China. *Frontiers in Plant Science*, *12*. https://doi.org/10.3389/fpls.2021.659130
28. Choudhary, D., Rawat, M., Mashkey, V. K., Sharma, V., & Kundu, P. (2025). Estimation of seaweed extract and micronutrient potential to improve net returns by enhancing yield characters in tomato using correlation analysis. *Journal of Applied Biology and Biotechnology*, *13*(1), 243–249. https://doi.org/10.7324/JABB.2024.199958
29. Chouliaras, V., Tasioula, M., Chatzissavvidis, C., Therios, I., & Tsabolatidou, E. (2009). The effects of a seaweed extract in addition to nitrogen and boron fertilization on productivity, fruit maturation, leaf nutritional status and oil quality of the olive (Olea europaea L.) cultivar Koroneiki. *Journal of the Science of Food and Agriculture*, *89*(6), 984–988. https://doi.org/10.1002/jsfa.3543
30. Ciepiela, G. A., & Godlewska, A. (2019a). The effect of biostimulants derived from various materials on the yield and selected organic components of italian rye grass (lolium multiflorum lam.) against the background of nitrogen regime. *Applied Ecology and Environmental Research*, *17*(5), 12407–12418. https://doi.org/10.15666/aeer/1705_1240712418
31. Ciepiela, G. A., & Godlewska, A. (2019b). The effect of biostimulants derived from various materials on the yield and selected organic components of italian rye grass (lolium multiflorum lam.) against the background of nitrogen regime. *Applied Ecology and Environmental Research*, *17*(5), 12407–12418. https://doi.org/10.15666/aeer/1705_1240712418
32. Colavita, G. M., Spera, N., Blackhall, V., & Sepulveda, G. M. (2011). Effect of seaweed extract on pear fruit quality and yield. *Acta Horticulturae*, *909*, 601–608. https://doi.org/10.17660/actahortic.2011.909.72
33. Colla, G., Cardarelli, M., Bonini, P., & Rouphael, Y. (2017). Foliar applications of protein hydrolysate, plant and seaweed extracts increase yield but differentially modulate fruit quality of greenhouse tomato. *HortScience*, *52*(9), 1214–1220. https://doi.org/10.21273/HORTSCI12200-17
34. Consentino, B. B., Vultaggio, L., Iacuzzi, N., la Bella, S., de Pasquale, C., Rouphael, Y., Ntatsi, G., Virga, G., & Sabatino, L. (2023). Iodine Biofortification and Seaweed Extract-Based Biostimulant Supply Interactively Drive the Yield, Quality, and Functional Traits in Strawberry Fruits. *Plants*, *12*(2). https://doi.org/10.3390/plants12020245
35. Consentino, B. B., Vultaggio, L., Sabatino, L., Ntatsi, G., Rouphael, Y., Bondì, C., de Pasquale, C., Guarino, V., Iacuzzi, N., Capodici, G., & Mauro, R. P. (2023). Combined effects of biostimulants, N level and drought stress on yield, quality and physiology of greenhouse-grown basil. *Plant Stress*, *10*. https://doi.org/10.1016/j.stress.2023.100268
36. Correia, S., Queirós, F., Ferreira, H., Morais, M. C., Afonso, S., Silva, A. P., & Gonçalves, B. (2020). Foliar application of calcium and growth regulators modulate sweet cherry (Prunus avium l.) tree performance. *Plants*, *9*(4). https://doi.org/10.3390/plants9040410
37. Cozzolino, E., di Mola, I., Ottaiano, L., Nocerino, S., Sifola, M. I., El-Nakhel, C., Rouphael, Y., & Mori, M. (2021a). Can seaweed extract improve yield and quality of brewing barley subjected to different levels of nitrogen fertilization? *Agronomy*, *11*(12). https://doi.org/10.3390/agronomy11122481
38. Cozzolino, E., di Mola, I., Ottaiano, L., Nocerino, S., Sifola, M. I., El-Nakhel, C., Rouphael, Y., & Mori, M. (2021b). Can seaweed extract improve yield and quality of brewing barley subjected to different levels of nitrogen fertilization? *Agronomy*, *11*(12). https://doi.org/10.3390/agronomy11122481
39. da Silva, B. A., Silva, J. de S., da Silva, T. I., da Costa, R. S., de Castro, C. S., de Oliveira, L. K. B., de Sousa, T. R. M., Rodrigues, C. Y. A. C., Cardoso, F. B., & Mesquita, R. O. (2024). Bioestimulant with Ascophyllum nodosum and fulvic acids as mitigating factors of salinity damage in soybean1. *Revista Brasileira de Engenharia Agricola e Ambiental*, *28*(4). https://doi.org/10.1590/1807-1929/agriambi.v28n4e278961
40. D’Addabbo, T., Laquale, S., Perniola, M., & Candido, V. (2019). Biostimulants for plant growth promotion and sustainable management of phytoparasitic nematodes in vegetable crops. *Agronomy*, *9*(10). https://doi.org/10.3390/agronomy9100616
41. Dalal, A., Bourstein, R., Haish, N., Shenhar, I., Wallach, R., & Moshelion, M. (2019). Dynamic physiological phenotyping of drought-stressed pepper plants treated with “productivity-enhancing” and “survivability-enhancing” biostimulants. *Frontiers in Plant Science*, *10*. https://doi.org/10.3389/fpls.2019.00905
42. de Carvalho, R. P., Pasqual, M., de Oliveira Silveira, H. R., de Melo, P. C., Bispo, D. F. A., Laredo, R. R., & de Aguiar Saldanha Lima, L. (2019). “Niágara Rosada” table grape cultivated with seaweed extracts: physiological, nutritional, and yielding behavior. *Journal of Applied Phycology*, *31*(3), 2053–2064. https://doi.org/10.1007/s10811-018-1724-7
43. de Clercq, P., Pauwels, E., Top, S., Steppe, K., & van Labeke, M. C. (2023). Effect of Seaweed-Based Biostimulants on Growth and Development of Hydrangea paniculata under Continuous or Periodic Drought Stress. *Horticulturae*, *9*(4). https://doi.org/10.3390/horticulturae9040509
44. Dehkordi, R. A., Roghani, S. R., Mafakheri, S., & Asghari, B. (2021). Effect of biostimulants on morpho-physiological traits of various ecotypes of fenugreek (Trigonella foenum-graecum L.) under water deficit stress. *Scientia Horticulturae*, *283*. https://doi.org/10.1016/j.scienta.2021.110077
45. Devi, H. S., Bokado, K., Barkha, Jackson, K., & Sonia. (2024). Enhancing growth and yield of the wheat-chickpea intercropping system through a combination of different row ratios and biostimulants. *Journal of Applied and Natural Science*, *16*(3), 1240–1249. https://doi.org/10.31018/jans.v16i3.5795
46. di Mola, I., Cozzolino, E., Ottaiano, L., Giordano, M., Rouphael, Y., El-Nakhel, C., Leone, V., & Mori, M. (2020). Effect of seaweed (Ecklonia maxima) extract and legume-derived protein hydrolysate biostimulants on baby leaf lettuce grown on optimal doses of nitrogen under greenhouse conditions. *Australian Journal of Crop Science*, *14*(9), 1456–1464. https://doi.org/10.21475/ajcs.20.14.09.p2511
47. di Stasio, E., Cirillo, V., Raimondi, G., Giordano, M., Esposito, M., & Maggio, A. (2020). Osmo-priming with seaweed extracts enhances yield of salt-stressed tomato plants. *Agronomy*, *10*(10 October). https://doi.org/10.3390/agronomy10101559
48. Di-Vaio, C., Cirillo, A., Cice, D., El-Nakhel, C., & Rouphael, Y. (2021). Biostimulant application improves yield parameters and accentuates fruit color of annurca apples. *Agronomy*, *11*(4). https://doi.org/10.3390/agronomy11040715
49. do Rosário Rosa, V., Farias dos Santos, A. L., Alves da Silva, A., Peduti Vicentini Sab, M., Germino, G. H., Barcellos Cardoso, F., & de Almeida Silva, M. (2021). Increased soybean tolerance to water deficiency through biostimulant based on fulvic acids and Ascophyllum nodosum (L.) seaweed extract. *Plant Physiology and Biochemistry*, *158*, 228–243. https://doi.org/10.1016/j.plaphy.2020.11.008
50. Dogra, B. S., & Mandradia, R. K. (2024). Effect of seaweed extract on growth and yield of onion. In *International Journal of Farm Sciences* (Vol. 164, Issue 17). www.IndianJournals.com
51. Domingo, G., Marsoni, M., Álvarez-Viñas, M., Torres, M. D., Domínguez, H., & Vannini, C. (2023). The Role of Protein-Rich Extracts from Chondrus crispus as Biostimulant and in Enhancing Tolerance to Drought Stress in Tomato Plants. *Plants*, *12*(4). https://doi.org/10.3390/plants12040845
52. Dudaš, S., Šola, I., Sladonja, B., Erhatić, R., Ban, D., & Poljuha, D. (2016). The effect of biostimulant and fertilizer on “Low Input” lettuce production. *Acta Botanica Croatica*, *75*(2), 253–259. https://doi.org/10.1515/botcro-2016-0023
53. Enan, S. A. A. M., El-Saady, A. M., & El-Sayed, A. B. (2016). Impact of Foliar Feeding With Alga Extract and Boron on Yield and Quality of Sugar Beet Grown in Sandy Soil. In *Egypt. J. Agron* (Vol. 38, Issue 2).
54. Espinosa-Antón, A. A., & Hernández-Herrera, R. M. (2024). Effects of green seaweed (Ulva onhoi) on the reproductive development of tomato (Solanum lycopersicum) plants. *Acta Agrobotanica*, *77*. <https://doi.org/10.5586/AA/193117>
55. Farouk, S., Youssef, S. A., Ali, A.A. (2012). Exploitation of bioestimulants and vitamins as an alternative strategy to control early blight of tomate plants. *Asian Journal of Plant Sciences,* 11(1), 36-43.
56. Frioni, T., Sabbatini, P., Tombesi, S., Norrie, J., Poni, S., Gatti, M., & Palliotti, A. (2018). Effects of a biostimulant derived from the brown seaweed Ascophyllum nodosum on ripening dynamics and fruit quality of grapevines. *Scientia Horticulturae*, *232*, 97–106. https://doi.org/10.1016/j.scienta.2017.12.054
57. Gandhi, G., Biswas, K., Vaghela, P., Nayak, J., Nair, A., Moradiya, K., Gopalakrishnan, V. A. K., Veeragurunathan, V., & Ghosh, A. (2024). In-depth metabolite characterization of seaweed-based plant biostimulants: Insights into bioactive components. *Algal Research*, *81*. https://doi.org/10.1016/j.algal.2024.103574
58. Głosek-Sobieraj, M., Cwalina-Ambroziak, B., & Hamouz, K. (2018). The Effect of Growth Regulators and a Biostimulator on the Health Status, Yield and Yield Components of Potatoes (Solanum tuberosum L.). *Gesunde Pflanzen*, *70*(1), 1–11. https://doi.org/10.1007/s10343-017-0407-7
59. Godlewska, A., & Anna Ciepiela, G. (2017). Effectiveness of fertilization of dactylis glomerata and festulolium braunii with nitrogen and the biostimulant kelpak sl. *34*. www.incda-fundulea.ro
60. Godlewska, A., & Ciepiela, G. A. (2016). The effect of growth regulator on dry matter yield and some chemical components in selected grass species and cultivars. *Soil Science and Plant Nutrition*, *62*(3), 297–302. https://doi.org/10.1080/00380768.2016.1185741
61. Godlewska, A., & Ciepiela, G. A. (2018). Assessment of the effect of various biostimulants on medicago X varia T. Martyn yielding and content of selected organic components. *Applied Ecology and Environmental Research*, *16*(5), 5571–5581. https://doi.org/10.15666/aeer/1605_55715581
62. Godlewska, A., & Ciepiela, G. A. (2020). Yield performance and content of selected organic compounds in Trifolium pratense treated with various biostimulants against the background of nitrogen fertilisation. *Legume Research*, *43*(6), 850–855. https://doi.org/10.18805/LR-522
63. Gómez, S., & Gómez, C. (2022). Evaluating the Use of Biostimulants for Indoor Hydroponic Lettuce Production. *HortTechnology*, *32*(4), 348–355. https://doi.org/10.21273/HORTTECH05045-22
64. Goñi, O., Łangowski, Ł., Feeney, E., Quille, P., & O’Connell, S. (2021). Reducing Nitrogen Input in Barley Crops While Maintaining Yields Using an Engineered Biostimulant Derived From Ascophyllum nodosum to Enhance Nitrogen Use Efficiency. *Frontiers in Plant Science*, *12*. https://doi.org/10.3389/fpls.2021.664682
65. Goñi, O., Quille, P., & O’Connell, S. (2018). Ascophyllum nodosum extract biostimulants and their role in enhancing tolerance to drought stress in tomato plants. *Plant Physiology and Biochemistry*, *126*, 63–73. https://doi.org/10.1016/j.plaphy.2018.02.024
66. González-González, M. F., Ocampo-Alvarez, H., Santacruz-Ruvalcaba, F., Sánchez-Hernández, C. V., Casarrubias-Castillo, K., Becerril-Espinosa, A., Castañeda-Nava, J. J., & Hernández-Herrera, R. M. (2020). Physiological, Ecological, and Biochemical Implications in Tomato Plants of Two Plant Biostimulants: Arbuscular Mycorrhizal Fungi and Seaweed Extract. *Frontiers in Plant Science*, *11*. https://doi.org/10.3389/fpls.2020.00999
67. Goyal, V., Kumari, A., Avtar, R., Baliyan, V., & Mehrotra, S. (2023). Orthosilicic acid and Seaweed Extract Alleviate the Deteriorative Effects of High Temperature Stress in Brassica juncea (L.) Czern & Coss. *Silicon*, *15*(11), 4909–4919. https://doi.org/10.1007/s12633-023-02376-9
68. Gugala, M., Zarzecka, K., Dolega, H., & Sikorska, A. (2018). Weed infestation and yielding of potato under conditions of varied use of herbicides and bio-stimulants. *Journal of Ecological Engineering*, *19*(4), 191–196. https://doi.org/10.12911/22998993/89654
69. Gupta, S., Stirk, W. A., Plačková, L., Kulkarni, M. G., Doležal, K., & van Staden, J. (2021). Interactive effects of plant growth-promoting rhizobacteria and a seaweed extract on the growth and physiology of Allium cepa L. (onion). *Journal of Plant Physiology*, *262*. https://doi.org/10.1016/j.jplph.2021.153437
70. Gurmani, Z. A., Khan, S., Khan, A., Farid, A., Khan, S., & Hameed, M. U. (2021). Optimization of Biostimulants Application for Phenology and Quality of Oats. *Brazilian Archives of Biology and Technology*, *64*. https://doi.org/10.1590/1678-4324-2021200726
71. Gyogluu Wardjomto, C., Mohammed, M., Ngmenzuma, T. Y., & Mohale, K. C. (2023). Effect of rhizobia inoculation and seaweed extract (Ecklonia maxima) application on the growth, symbiotic performance and nutritional content of cowpea (Vigna unguiculata (L.) Walp.). *Frontiers in Agronomy*, *5*. https://doi.org/10.3389/fagro.2023.1138263
72. Hernández-Herrera, R. M., Sánchez-Hernández, C. V., Palmeros-Suárez, P. A., Ocampo-Alvarez, H., Santacruz-Ruvalcaba, F., Meza-Canales, I. D., & Becerril-Espinosa, A. (2022). Seaweed Extract Improves Growth and Productivity of Tomato Plants under Salinity Stress. *Agronomy*, *12*(10). <https://doi.org/10.3390/agronomy12102495>
73. Hidangmayun, A., Sharma, R. (2017). Effect of different concentrations of commercial seaweed liquid extract of Ascophyllum nodosum as a plant bio stimulant on growth, yield and biochemical constituents of onion (Allium cepa L.). Journal of Pharmacognosy and Phytochemistry, 6(4), 658-663.
74. Jacomassi, L. M., Viveiros, J. de O., Oliveira, M. P., Momesso, L., de Siqueira, G. F., & Crusciol, C. A. C. (2022). A Seaweed Extract-Based Biostimulant Mitigates Drought Stress in Sugarcane. *Frontiers in Plant Science*, *13*. https://doi.org/10.3389/fpls.2022.865291
75. Jadhao, G. R., Chaudhary, D. R., Khadse, V. A., & Zodape, S. T. (2015). Utilization of seaweeds in enhancing productivity and quality of black gram [Vigna mungo (L.) Hepper] for sustainable agriculture. In *Indian Journal of Natural Products and Resources* (Vol. 6, Issue 1).
76. Jannin, L., Arkoun, M., Etienne, P., Laîné, P., Goux, D., Garnica, M., Fuentes, M., Francisco, S. S., Baigorri, R., Cruz, F., Houdusse, F., Garcia-Mina, J. M., Yvin, J. C., & Ourry, A. (2013). Brassica napus Growth is Promoted by Ascophyllum nodosum (L.) Le Jol. Seaweed Extract: Microarray Analysis and Physiological Characterization of N, C, and S Metabolisms. *Journal of Plant Growth Regulation*, *32*(1), 31–52. https://doi.org/10.1007/s00344-012-9273-9
77. Kalozoumis, P., Vourdas, C., Ntatsi, G., & Savvas, D. (2021). Can biostimulants increase resilience of hydroponically-grown tomato to combined water and nutrient stress? *Horticulturae*, *7*(9). https://doi.org/10.3390/horticulturae7090297
78. Kanojia, A., Lyall, R., Sujeeth, N., Alseekh, S., Martínez-Rivas, F. J., Fernie, A. R., Gechev, T. S., & Petrov, V. (2024). Physiological and molecular insights into the effect of a seaweed biostimulant on enhancing fruit yield and drought tolerance in tomato. *Plant Stress*, *14*. https://doi.org/10.1016/j.stress.2024.100692
79. Karthik, T., & Jayasri, M. A. (2023a). Exploration of biostimulant property of seaweed liquid extract (SLE) on the growth development and yield of Solanum lycopersicum (Tomato). *Aquaculture International*, *31*(6), 3189–3205. https://doi.org/10.1007/s10499-023-01252-y
80. Karthik, T., & Jayasri, M. A. (2023b). Systematic study on the effect of seaweed fertilizer on the growth and yield of Vigna radiata (L.) R. Wilczek (Mung bean). *Journal of Agriculture and Food Research*, *14*. https://doi.org/10.1016/j.jafr.2023.100748
81. Karthikeyan, K., & Shanmugam, M. (2017). The effect of potassium-rich biostimulant from seaweed Kappaphycus alvarezii on yield and quality of cane and cane juice of sugarcane var. Co 86032 under plantation and ratoon crops. *Journal of Applied Phycology*, *29*(6), 3245–3252. https://doi.org/10.1007/s10811-017-1211-6
82. Khan, Z., Gul, H., Rauf, M., Arif, M., Hamayun, M., Ud-Din, A., Sajid, Z. A., Khilji, S. A., Rehman, A., Tabassum, A., Parveen, Z., & Lee, I. J. (2022). Sargassum wightii Aqueous Extract Improved Salt Stress Tolerance in Abelmoschus esculentus by Mediating Metabolic and Ionic Rebalance. *Frontiers in Marine Science*, *9*. https://doi.org/10.3389/fmars.2022.853272
83. Knapowski, T., Barczak, B., Kozera, W., Wszelaczyńska, E., & Poberezny, J. (2019). Crop stimulants as a factor determining the yield and quality of winter wheat grown in Notec Valley, Poland. *Current Science*, *116*(6), 1009–1015. https://doi.org/10.18520/cs/v116/i6/1009-1015
84. Kocira, A., Lamorska, J., Kornas, R., Nowosad, N., Tomaszewska, M., Leszczyńska, D., Kozłowicz, K., & Tabor, S. (2020). Changes in biochemistry and yield in response to biostimulants applied in bean (Phaseolus vulgaris L.). *Agronomy*, *10*(2). https://doi.org/10.3390/agronomy10020189
85. Kocira, S., Szparaga, A., Findura, P., & Treder, K. (2020a). Modification of yield and fiber fractions biosynthesis in phaseolus vulgaris by treatment with biostimulants containing amino acids and seaweed extract. *Agronomy*, *10*(9). https://doi.org/10.3390/agronomy10091338
86. Kocira, S., Szparaga, A., Findura, P., & Treder, K. (2020b). Modification of yield and fiber fractions biosynthesis in phaseolus vulgaris by treatment with biostimulants containing amino acids and seaweed extract. *Agronomy*, *10*(9). https://doi.org/10.3390/agronomy10091338
87. Kocira, S., Szparaga, A., Kuboń, M., Czerwińska, E., & Piskier, T. (2019). Morphological and biochemical responses of Glycine max (L.) Merr. To the use of seaweed extract. *Agronomy*, *9*(2). https://doi.org/10.3390/agronomy9020093
88. Kostadinova, S., Kalinova, S., Hristoskov, A., & Samodova, A. (2015). EfficiEncy of SomE foliar fErtilizErS in WintEr WhEat746 Agricultural Academy. In *Bulgarian Journal of Agricultural Science* (Vol. 21, Issue 4).
89. Krawczuk, A., Huyghebaert, B., Rabier, F., Parafiniuk, S., Przywara, A., Koszel, M., Lorencowicz, E., & Kocira, S. (2023). The Technical Parameters of Seaweed Biostimulant Spray Application as a Factor in the Economic Viability of Soybean Production. *Applied Sciences (Switzerland)*, *13*(2). https://doi.org/10.3390/app13021051
90. Kumar, R., Trivedi, K., Anand, K. G. V., & Ghosh, A. (2020). Science behind biostimulant action of seaweed extract on growth and crop yield: insights into transcriptional changes in roots of maize treated with Kappaphycus alvarezii seaweed extract under soil moisture stressed conditions. *Journal of Applied Phycology*, *32*(1), 599–613. https://doi.org/10.1007/s10811-019-01938-y
91. la Bella, S., Consentino, B. B., Rouphael, Y., Ntatsi, G., de Pasquale, C., Iapichino, G., & Sabatino, L. (2021). Impact of ecklonia maxima seaweed extract and mo foliar treatments on biofortification, spinach yield, quality and nue. *Plants*, *10*(6). https://doi.org/10.3390/plants10061139
92. Lakshmi, S., Ravichandran, V., Anandakumar, S., Senthil, A., Arul, L., Radhamani, S., & Anupriya, R. (2023). Foliar application of Ascophyllum nodosum on improvement of photosynthesis, fruit setting percentage, yield and quality of tomato (Solanum lycopersicum L.). *Journal of Applied and Natural Science*, *15*(3), 961–971. https://doi.org/10.31018/jans.v15i3.4725
93. Łangowski, Ł., Goñi, O., Marques, F. S., Hamawaki, O. T., da Silva, C. O., Nogueira, A. P. O., Teixeira, M. A. J., Glasenapp, J. S., Pereira, M., & O’Connell, S. (2021). Ascophyllum nodosum Extract (SealicitTM) Boosts Soybean Yield Through Reduction of Pod Shattering-Related Seed Loss and Enhanced Seed Production. *Frontiers in Plant Science*, *12*. https://doi.org/10.3389/fpls.2021.631768
94. Łangowski, Ł., Goñi, O., Quille, P., Stephenson, P., Carmody, N., Feeney, E., Barton, D., Østergaard, L., & O’Connell, S. (2019). A plant biostimulant from the seaweed Ascophyllum nodosum (Sealicit) reduces podshatter and yield loss in oilseed rape through modulation of IND expression. *Scientific Reports*, *9*(1). https://doi.org/10.1038/s41598-019-52958-0
95. Layek, J., Das, A., Ramkrushna, G. I., Trivedi, K., Yesuraj, D., Chandramohan, M., Kubavat, D., Agarwal, P. K., & Ghosh, A. (2015). Seaweed sap: a sustainable way to improve productivity of maize in North-East India. *International Journal of Environmental Studies*, *72*(2), 305–315. <https://doi.org/10.1080/00207233.2015.1010855>
96. Lola-Luz, T., Hennequart, F., Gaffney, M. (2014). Effect on yield, total phenolic, total flavonoid and total isothiocyanate content of two broccoli cultivars (*Brassica oleraceae var italica*) following the application of a commercial brown seaweed extract (*Ascophyllum nodosum*). Agricultural and food science, 23, 28-37.  <https://doi.org/10.23986/afsci.8832>
97. López-Mosquera, M. E., & Pazos, P. (1997). Effects of Seaweed on Potato Yields and Soil Chemistry. *Biological Agriculture and Horticulture*, *14*(3), 199–205. https://doi.org/10.1080/01448765.1997.9754810
98. Mattner, S. W., Milinkovic, M., & Arioli, T. (2018). Increased growth response of strawberry roots to a commercial extract from Durvillaea potatorum and Ascophyllum nodosum. *Journal of Applied Phycology*, *30*(5), 2943–2951. https://doi.org/10.1007/s10811-017-1387-9
99. Mattner, S. W., Villalta, O. N., McFarlane, D. J., Islam, M. T., Arioli, T., & Cahill, D. M. (2023). The biostimulant effect of an extract from Durvillaea potatorum and Ascophyllum nodosum is associated with the priming of reactive oxygen species in strawberry in south-eastern Australia. *Journal of Applied Phycology*, *35*(4), 1789–1800. https://doi.org/10.1007/s10811-023-02979-0
100. Matysiak, K., Miziniak, W., Kaczmarek, S., & Kierzek, R. (2018). Herbicides with natural and synthetic biostimulants in spring wheat. *Ciencia Rural*, *48*(11). https://doi.org/10.1590/0103-8478cr20180405
101. Mazepa, E., Malburg, B. v., Mógor, G., de Oliveira, A. C., Amatussi, J. O., Corrêa, D. O., Lemos, J. S., Ducatti, D. R. B., Duarte, M. E. R., Mógor, Á. F., & Noseda, M. D. (2021). Plant growth biostimulant activity of the green microalga Desmodesmus subspicatus. *Algal Research*, *59*. https://doi.org/10.1016/j.algal.2021.102434
102. Merwad, A. R. M. A. (2020). Mitigation of salinity stress effects on growth, yield and nutrient uptake of wheat by application of organic extracts. *Communications in Soil Science and Plant Analysis*, *51*(9), 1150–1160. https://doi.org/10.1080/00103624.2020.1751188
103. Mesara, S., Akhyani, D. D., Agarwal, P., Gangapur, D. R., & Agarwal, P. K. (2024). Sargassum tenerrimum extract reduces Sclerotium rolfsii stem rot disease in peanut by modulating physio-biochemical responses. *Acta Physiologiae Plantarum*, *46*(7). https://doi.org/10.1007/s11738-024-03697-x
104. Meyer, F. R., Orioli Júnior, V., Bernardes, J. V. S., & Coelho, V. P. D. M. (2021). Foliar spraying of a seaweed-based biostimulant in soybean. *Revista Caatinga*, *34*(1), 99–107. https://doi.org/10.1590/1983-21252021v34n111rc
105. Michalak, I., Chojnacka, K., Dmytryk, A., Wilk, R., Gramza, M., & Rój, E. (2016). Evaluation of supercritical extracts of algae as biostimulants of plant growth in field trials. *Frontiers in Plant Science*, *7*(OCTOBER2016). https://doi.org/10.3389/fpls.2016.01591
106. Michalak, I., Wilk, R., & Chojnacka, K. (2017). Bioconversion of Baltic Seaweeds into Organic Compost. *Waste and Biomass Valorization*, *8*(6), 1885–1895. https://doi.org/10.1007/s12649-016-9738-3
107. Mola, I. di, Cozzolino, E., Ottaiano, L., Giordano, M., Rouphael, Y., Colla, G., & Mori, M. (2019). Effect of vegetal- And seaweed extract-based biostimulants on agronomical and leaf quality traits of plastic tunnel-grown baby lettuce under four regimes of nitrogen fertilization. *Agronomy*, *9*(10). https://doi.org/10.3390/agronomy9100571
108. Mousavi, S. M., Jafari, A., & Shirmardi, M. (2024). The effect of seaweed foliar application on yield and quality of apple cv. ‘Golden Delicious.’ *Scientia Horticulturae*, *323*. https://doi.org/10.1016/j.scienta.2023.112529
109. Munisamy, S., & Ramamoorthy, G. K. (2024). Response of Cassava Root Manihot esculenta to Potassium-Rich Biostimulants Manufactured from Red Seaweed Gracilaria salicornia Under Semi-Arid Condition. *Agricultural Research*, *13*(3), 484–497. https://doi.org/10.1007/s40003-024-00717-7
110. Naz, S., Muhammad, H. M. D., Ramzan, M., Sadiq, B., Ahmad, R., Ali, S., Alsahli, A. A., & Altaf, M. A. (2023). Seaweed Application Enhanced the Growth and Yield of Pea (Pisum sativum L.) by Altering Physiological Indices. *Journal of Soil Science and Plant Nutrition*, *23*(4), 6183–6195. https://doi.org/10.1007/s42729-023-01475-1
111. Norrie, J., Branson, T., & Keathley, P. E. (n.d.). *Marine Plant Extracts Impact on grape Yield and Quality*.
112. Oñez, L. J. P., Catubis, K. M. L., Cabillo, R. A., & Pascual, P. R. L. (2024). Improved morphological and yield responses of green lettuce (Lactuca sativa L.) grown with seaweed extract as a hydroponic nutrient solution. Journal of Plant Nutrition, 48(4), 658–669. <https://doi.org/10.1080/01904167.2024.2411402>
113. Osman, H. S., Gamal, H., El-Gawad, A., & Osman, H. S. (2014). Effect of Exogenous Application of Boric Acid and Seaweed Extract on Growth, Biochemical Content and Yield of Eggplant. *Journal of Horticultural Science & Ornamental Plants*, *6*(3), 133–143. https://doi.org/10.5829/idosi.jhsop.2014.6.3.1147
114. Ozbay, N., & Demirkiran, A. R. (2019). Enhancement of growth in ornamental pepper (Capsicum Annuum L.) plants with application of a commercial seaweed product, stimplex®. *Applied Ecology and Environmental Research*, *17*(2), 4361–4375. https://doi.org/10.15666/aeer/1702_43614375
115. Pačuta, V., Rašovský, M., Briediková, N., Lenická, D., Ducsay, L., & Zapletalová, A. (2023a). *Plant Biostimulants as an Effective Tool for Increasing Physiological Activity and Productivity of Different Sugar Beet Varieties*. https://doi.org/10.3390/agronomy
116. Pačuta, V., Rašovský, M., Briediková, N., Lenická, D., Ducsay, L., & Zapletalová, A. (2023b). *Plant Biostimulants as an Effective Tool for Increasing Physiological Activity and Productivity of Different Sugar Beet Varieties*. https://doi.org/10.3390/agronomy
117. Paglialunga, G., Proietti, S., Cardarelli, M., Moscatello, S., Colla, G., & Battistelli, A. (2022). Chicory Taproot Production: Effects of Biostimulants under Partial or Full Controlled Environmental Conditions. *Agronomy*, *12*(11). https://doi.org/10.3390/agronomy12112816
118. Petoumenou, D. G., & Patris, V. E. (2021). Effects of several preharvest canopy applications on yield and quality of table grapes (Vitis vinifera l.) cv. crimson seedless. *Plants*, *10*(5). https://doi.org/10.3390/plants10050906
119. Petropoulos, S. A., Fernandes, Â., Plexida, S., Chrysargyris, A., Tzortzakis, N., Barreira, J. C. M., Barros, L., & Ferreira, I. C. F. R. (2020). Biostimulants application alleviates water stress effects on yield and chemical composition of greenhouse green bean (phaseolus vulgaris l.). *Agronomy*, *10*(2). https://doi.org/10.3390/agronomy10020181
120. Pohl, A., Grabowska, A., Kalisz, A., & Sekara, A. (2018). Preliminary screening of biostimulative effects of Göemar BM-86 on eggplant cultivars grown under field conditions in Poland. *Acta Agrobotanica*, *71*(4). https://doi.org/10.5586/aa.1752
121. Pohl, A., Grabowska, A., Kalisz, A., & Sȩkara, A. (2019). The eggplant yield and fruit composition as affected by genetic factor and biostimulant application. *Notulae Botanicae Horti Agrobotanici Cluj-Napoca*, *47*(3), 929–938. https://doi.org/10.15835/nbha47311468
122. Polo, J., & Mata, P. (2018). Evaluation of a biostimulant (Pepton) based in enzymatic hydrolyzed animal protein in comparison to seaweed extracts on root development, vegetative growth, flowering, and yield of gold cherry tomatoes grown under low stress ambient field conditions. *Frontiers in Plant Science*, *8*. https://doi.org/10.3389/fpls.2017.02261
123. Prokkola, S., & Kivijärvi, P. (2007). *Effect of biological sprays on the incidence of grey mould, fruit yield and fruit quality in organic strawberry production* (Vol. 16).
124. Quassi de Castro, S. G., de Castro, S. A. Q., de Castro, R. A., de Castro, R. R. L., Dorante, L. T., Souza, R. S., & Hippler, F. W. R. (2024). Combining seaweed extract from Ascophyllum nodosum with nutrients enhances stalk yield when applied in the dry season during sugarcane development. *Plant Stress*, *13*. https://doi.org/10.1016/j.stress.2024.100535
125. Quille, P., Claffey, A., Feeney, E., Kacprzyk, J., Ng, C. K. Y., & O’connell, S. (2022). The Effect of an Engineered Biostimulant Derived from Ascophyllum nodosum on Grass Yield under a Reduced Nitrogen Regime in an Agronomic Setting. *Agronomy*, *12*(2). https://doi.org/10.3390/agronomy12020463
126. Raj, Y., Ali, N., Pati, A. M., & Kumar, R. (2022). Cleaner production technologies for the amelioration of soil health, biomass and secondary metabolites in Ocimum basilicum L. under Indian Western Himalaya. *Frontiers in Plant Science*, *13*. https://doi.org/10.3389/fpls.2022.976295
127. Raju, G., Sri Sailaja, N., Krishnapriya, V., & Prakash, &. (2022). Ameliorating drought stress in sugarcane (Saccharum spp.) using biostimulants. In *Indian Journal of Experimental Biology* (Vol. 60).
128. Rakian, T. C., Kilowasid, L. M. H., Afa, L. O., Riskyana, A., Nurazizah, Wijayanti, Y., Bahrun, A., Subair, I., Rahni, N. M., Alam, S., Sarawa, & Karimuna, L. (2023). Soil biological quality in rhizosphere, growth, and yield of upland rice grown on acid soil after amended biochar enriched sap of Kappaphycus alvarezii. *Biodiversitas*, *24*(12), 6780–6792. https://doi.org/10.13057/biodiv/d241241
129. Rana, V. S., Lingwal, K., Sharma, S., Rana, N., Pawar, R., Kumar, V., & Sharma, U. (2023). Enhancement in growth, yield and nutritive characteristics of strawberry (Fragaria × ananassa Duch.) by the application of biostimulant: seaweed extract. *Acta Physiologiae Plantarum*, *45*(10). https://doi.org/10.1007/s11738-023-03602-y
130. Rana, V. S., Sharma, V., Sharma, S., Rana, N., Kumar, V., Sharma, U., Almutairi, K. F., Avila-Quezada, G. D., Abd_Allah, E. F., & Gudeta, K. (2023). Seaweed Extract as a Biostimulant Agent to Enhance the Fruit Growth, Yield, and Quality of Kiwifruit. *Horticulturae*, *9*(4). https://doi.org/10.3390/horticulturae9040432
131. Rasouli, F., Amini, T., Asadi, M., Hassanpouraghdam, M. B., Aazami, M. A., Ercisli, S., Skrovankova, S., & Mlcek, J. (2022). Growth and Antioxidant Responses of Lettuce (Lactuca sativa L.) to Arbuscular Mycorrhiza Inoculation and Seaweed Extract Foliar Application. *Agronomy*, *12*(2). https://doi.org/10.3390/agronomy12020401
132. Rathinapriya, P., Satish, L., Pandian, S., Rameshkumar, R., Balasangeetha, M., Rakkammal, K., & Ramesh, M. (2020). Effects of liquid seaweed extracts in improving the agronomic performance of foxtail millet. *Journal of Plant Nutrition*, *43*(19), 2857–2875. https://doi.org/10.1080/01904167.2020.1799002
133. Rathore, S. S., Chaudhary, D. R., Boricha, G. N., Ghosh, A., Bhatt, B. P., Zodape, S. T., & Patolia, J. S. (2009). Effect of seaweed extract on the growth, yield and nutrient uptake of soybean (Glycine max) under rainfed conditions. *South African Journal of Botany*, *75*(2), 351–355. https://doi.org/10.1016/j.sajb.2008.10.009
134. Raverkar, K. P., Pareek, N., Chandra, R., Chauhan, S., Zodape, S. T., & Ghosh, A. (2016). Impact of foliar application of seaweed saps on yield, nodulation and nutritional quality in green gram (Vigna radiata L). *Legume Research*, *39*(2), 315–318. https://doi.org/10.18805/lr.v39i2.9535
135. Renaut, S., Masse, J., Norrie, J. P., Blal, B., & Hijri, M. (2019). A commercial seaweed extract structured microbial communities associated with tomato and pepper roots and significantly increased crop yield. *Microbial Biotechnology*, *12*(6), 1346–1358. https://doi.org/10.1111/1751-7915.13473
136. Rossini, A., Ruggeri, R., & Rossini, F. (2024). Discriminating among Alternative Dressing Solutions for Cereal Seed Treatment: Effect on Germination and Seedling Vigor of Durum Wheat. *International Journal of Plant Biology*, *15*(2), 230–241. https://doi.org/10.3390/ijpb15020019
137. Rouphael, Y., Giordano, M., Cardarelli, M., Cozzolino, E., Mori, M., Kyriacou, M. C., Bonini, P., & Colla, G. (2018a). Plant-and seaweed-based extracts increase yield but differentially modulate nutritional quality of greenhouse spinach through biostimulant action. *Agronomy*, *8*(7). https://doi.org/10.3390/agronomy8070126
138. Rouphael, Y., Giordano, M., Cardarelli, M., Cozzolino, E., Mori, M., Kyriacou, M. C., Bonini, P., & Colla, G. (2018b). Plant-and seaweed-based extracts increase yield but differentially modulate nutritional quality of greenhouse spinach through biostimulant action. *Agronomy*, *8*(7). https://doi.org/10.3390/agronomy8070126
139. Ruban, S., Priya, M. R., Barathan, G., & Suresh Kumar, S. M. (n.d.). *Effect of foliar application of biostimulants on yield of brinjal (Solanum melongena L.)*.
140. Sabir, A., Yazar, K., Sabir, F., Kara, Z., Yazici, M. A., & Goksu, N. (2014). Vine growth, yield, berry quality attributes and leaf nutrient content of grapevines as influenced by seaweed extract (Ascophyllum nodosum) and nanosize fertilizer pulverizations. *Scientia Horticulturae*, *175*, 1–8. https://doi.org/10.1016/j.scienta.2014.05.021
141. Salvage, R., Cannon, T., Kingsmill, P., Liu, F., & Fleming, C. C. (2024). A complex biostimulant based on plant flavonoids enhances potato growth and commercial yields. *Frontiers in Sustainable Food Systems*, *8*. https://doi.org/10.3389/fsufs.2024.1368423
142. Sandhu, R. K., Nandwani, D., & Nwosisi, S. (2018). Assessing seaweed extract as a biostimulant on the yield of organic leafy greens in Tennessee. In *J. Agric. Univ. P.R* (Vol. 102, Issue 2).
143. Santoro, D. F., Puglisi, I., Sicilia, A., Baglieri, A., la Bella, E., & lo Piero, A. R. (2023). Transcriptomic profile of lettuce seedlings (Lactuca sativa) response to microalgae extracts used as biostimulant agents. *AoB PLANTS*, *15*(4). https://doi.org/10.1093/aobpla/plad043
144. Santos, P. L. F. dos, Zabotto, A. R., Jordão, H. W. C., Boas, R. L. V., Broetto, F., & Tavares, A. R. (2019). Use of seaweed-based biostimulant (Ascophyllum nodosum) on ornamental sunflower seed germination and seedling growth. *Ornamental Horticulture*, *25*(3), 231–237. https://doi.org/10.1590/2447-536X.v25i3.2044
145. Seğmen, E., & Özdamar Ünlü, H. (2023). Effects of foliar applications of commercial seaweed and spirulina platensis extracts on yield and fruit quality in pepper (Capsicum annuum L.). *Cogent Food and Agriculture*, *9*(1). https://doi.org/10.1080/23311932.2023.2233733
146. Shah, M. T., Zodape, S. T., Chaudhary, D. R., Eswaran, K., & Chikara, J. (2013). Seaweed sap as an alternative liquid fertilizer for yield and quality improvement of wheat. *Journal of Plant Nutrition*, *36*(2), 192–200. https://doi.org/10.1080/01904167.2012.737886
147. Shahzad, R., Harlina, P. W., Gallego, P. P., Flexas, J., Ewas, M., Leiwen, X., & Karuniawan, A. (2023). The seaweed Ascophyllum nodosum-based biostimulant enhances salt stress tolerance in rice (Oryza sativa L.) by remodeling physiological, biochemical, and metabolic responses. *Journal of Plant Interactions*, *18*(1). https://doi.org/10.1080/17429145.2023.2266514
148. Shalaby, T. A., Ragab El-Ramady, H., & El-Ramady, H. (2014). Effect of foliar application of bio-stimulants on growth, yield components, and storability of garlic (Allium sativum L.) Effect of foliar application of bio-stimulants on growth, yield, components, and storability of garlic (Allium sativum L.). In *Article in Australian Journal of Crop Science* (Vol. 8, Issue 2). https://www.researchgate.net/publication/260603191
149. Sharma, L., Banerjee, M., Malik, G. C., Gopalakrishnan, V. A. K., Zodape, S. T., & Ghosh, A. (2017). Sustainable agro-technology for enhancement of rice production in the red and lateritic soils using seaweed based biostimulants. *Journal of Cleaner Production*, *149*, 968–975. <https://doi.org/10.1016/j.jclepro.2017.02.153>
150. Sharma S, Chen C, Khatri K, Rathore MS, Pandey SP. (2019). Gracilaria dura extract confers drought tolerance in wheat by modulating abscisic acid homeostasis. *Plant Physiol Biochem*. 136:143-154. doi: 10.1016/j.plaphy.2019.01.015.
151. Singh, I., Anand, K. G. V., Solomon, S., Shukla, S. K., Rai, R., Zodape, S. T., & Ghosh, A. (2018). Can we not mitigate climate change using seaweed based biostimulant: A case study with sugarcane cultivation in India. *Journal of Cleaner Production*, *204*, 992–1003. https://doi.org/10.1016/j.jclepro.2018.09.070
152. Singh, I., Solomon, S., Gopalakrishnan, V. A. K., & Ghosh, A. (2023). Environmental benefits of an alternative practice for sugarcane cultivation using Gracilaria-based seaweed biostimulant. *Sugar Tech*, *25*(2), 440–452. https://doi.org/10.1007/s12355-022-01217-0
153. Singh, P., Pattanaik, S., Jnanesha, A. C., Sunkari, R. K., Bhattarai, S., Varshney, V. K., Bharathkumar, S., & Lal, R. K. (2024). Augmenting root biomass productivity and reserpine level in Rauvolfia serpentina (L.) Benth. ex Kurz by foliar application of seaweed extracts. *Industrial Crops and Products*, *218*. https://doi.org/10.1016/j.indcrop.2024.118857
154. Soltaniband, V., Brégard, A., Gaudreau, L., & Dorais, M. (2022). Biostimulants Promote Plant Development, Crop Productivity, and Fruit Quality of Protected Strawberries. *Agronomy*, *12*(7). https://doi.org/10.3390/agronomy12071684
155. Soppelsa, S., Kelderer, M., Casera, C., Bassi, M., Robatscher, P., & Andreotti, C. (2018). Use of biostimulants for organic apple production: effects on tree growth, yield, and fruit quality at harvest and during storage. *Frontiers in Plant Science*, *9*. https://doi.org/10.3389/fpls.2018.01342
156. Soppelsa, S., Kelderer, M., Casera, C., Bassi, M., Robatscher, P., Matteazzi, A., & Andreotti, C. (2019). Foliar applications of biostimulants promote growth, yield and fruit quality of strawberry plants grown under nutrient limitation. *Agronomy*, *9*(9). https://doi.org/10.3390/agronomy9090483
157. Soppelsa, S., Kelderer, M., Testolin, R., Zanotelli, D., & Andreotti, C. (2020). Effect of biostimulants on apple quality at harvest and after storage. *Agronomy*, *10*(8). https://doi.org/10.3390/agronomy10081214
158. Sousa, F., Martins, M., Sousa, B., Soares, C., Azenha, M., Pereira, R., & Fidalgo, F. (2022). The potential of beach wrack as plant biostimulant to mitigate metal toxicity: mineral composition, antioxidant properties and effects against Cu-induced stress. *Journal of Applied Phycology*, *34*(1), 667–678. https://doi.org/10.1007/s10811-021-02636-4
159. Spinelli, F., Fiori, G., Noferini, M., Sprocatti, M., & Costa, G. (2009). Perspectives on the use of a seaweed extract to moderate the negative effects of alternate bearing in apple trees. *Journal of Horticultural Science and Biotechnology*, *84*(6), 131–137. https://doi.org/10.1080/14620316.2009.11512610
160. Stamatiadis, S., Evangelou, E., Jamois, F., & Yvin, J.-C. (n.d.-a). *Targeting Ascophyllum nodosum (L.) Le Jol. extract application at five growth stages of winter wheat*. https://doi.org/10.1007/s10811-021-02417-z/Published
161. Stamatiadis, S., Evangelou, E., Jamois, F., & Yvin, J.-C. (n.d.-b). *Targeting Ascophyllum nodosum (L.) Le Jol. extract application at five growth stages of winter wheat*. https://doi.org/10.1007/s10811-021-02417-z/Published
162. Subramaniyan, L., Veerasamy, R., Prabhakaran, J., Selvaraj, A., Algarswamy, S., Karuppasami, K. M., Thangavel, K., & Nalliappan, S. (2023). Biostimulation Effects of Seaweed Extract (Ascophyllum nodosum) on Phytomorpho-Physiological, Yield, and Quality Traits of Tomato (Solanum lycopersicum L.). *Horticulturae*, *9*(3). https://doi.org/10.3390/horticulturae9030348
163. Sulakhudin, Hatta, M., & Suryadi, U. E. (2019). Application of coastal sediments and foliar seaweed extract and its influence to soil properties, growth and yield of shallot in peatland. *Agrivita*, *41*(3), 450–460. https://doi.org/10.17503/agrivita.v41i3.939
164. Szczepanek, M. (2018). Technology of maize with growth stimulants application. *Engineering for Rural Development*, *17*, 483–490. https://doi.org/10.22616/ERDev2018.17.N074
165. Szczepanek, M., Jas´kiewicz, B., & Kotwica, K. (2018). Response of barley on seaweed biostimulant application. *Research for Rural Development*, *2*, 49–54. https://doi.org/10.22616/rrd.24.2018.050
166. Szczepanek, M., Wilczewski, E., Pobereżny, J., Wszelaczyńska, E., & Ochmian, I. (2017). Carrot root size distribution in response to biostimulant application. *Acta Agriculturae Scandinavica Section B: Soil and Plant Science*, *67*(4), 334–339. https://doi.org/10.1080/09064710.2017.1278783
167. Szczepanek, M., Wszelaczyńska, E., & Pobereżny, J. (2018). EFFECT OF SEAWEED BIOSTIMULANT APPLICATION IN SPRING WHEAT. *AgroLife Scientific Journal*, *7*(1).
168. Szo, I., Wieniarska, J. (2012) Effect of foliar applications of goëmar® bm 86 and soil applied calcium nitrate on yield and berry quality of two blue honeysuckle cultivars. *Acta Scientiarum Polonorum Hortorum Cultus,* 11 (1).
169. Szo, I.,
170. Tandon, S., & Dubey, A. (2015). Effects of Biozyme (Ascophyllum nodosum) Biostimulant on Growth and Development of Soybean [Glycine Max (L.) Merill]. *Communications in Soil Science and Plant Analysis*, *46*(7), 861–874. https://doi.org/10.1080/00103624.2015.1011749
171. Taskos, D., Stamatiadis, S., Yvin, J. C., & Jamois, F. (2019). Effects of an Ascophyllum nodosum (L.) Le Jol. extract on grapevine yield and berry composition of a Merlot vineyard. *Scientia Horticulturae*, *250*, 27–32. https://doi.org/10.1016/j.scienta.2019.02.030
172. TF, E.-S., SF, E.-G., & OA, A. (2015). Effect of Foliar Application with Algae and Plant Extracts on Growth, Yield and Fruit Quality of Fruitful Mango Trees Cv. Fagri Kalan. *Journal of Horticulture*, *02*(04). https://doi.org/10.4172/2376-0354.1000162
173. Trivedi, K., Anand, K. G. V., Kubavat, D., & Ghosh, A. (2022). Role of Kappaphycus alvarezii seaweed extract and its active constituents, glycine betaine, choline chloride, and zeatin in the alleviation of drought stress at critical growth stages of maize crop. *Journal of Applied Phycology*, *34*(3), 1791–1804. https://doi.org/10.1007/s10811-022-02722-1
174. Trivedi, K., Gopalakrishnan, V. A. K., Kumar, R., & Ghosh, A. (2021). Transcriptional Analysis of Maize Leaf Tissue Treated With Seaweed Extract Under Drought Stress. *Frontiers in Sustainable Food Systems*, *5*. https://doi.org/10.3389/fsufs.2021.774978
175. Trivedi, K., Kumar, R., Vijay Anand, K. G., Bhojani, G., Kubavat, D., & Ghosh, A. (2022). Structural and functional changes in soil bacterial communities by drifting spray application of a commercial red seaweed extract as revealed by metagenomics. *Archives of Microbiology*, *204*(1). https://doi.org/10.1007/s00203-021-02644-5
176. Trivedi, K., Vijay Anand, K. G., Kubavat, D., Kumar, R., Vaghela, P., & Ghosh, A. (2017). Crop stage selection is vital to elicit optimal response of maize to seaweed bio-stimulant application. *Journal of Applied Phycology*, *29*(4), 2135–2144. https://doi.org/10.1007/s10811-017-1118-2
177. Trivedi, K., Vijay Anand, K. G., Kubavat, D., Patidar, R., & Ghosh, A. (2018). Drought alleviatory potential of Kappaphycus seaweed extract and the role of the quaternary ammonium compounds as its constituents towards imparting drought tolerance in Zea mays L. *Journal of Applied Phycology*, *30*(3), 2001–2015. https://doi.org/10.1007/s10811-017-1375-0
178. Trivedi, K., Vijay Anand, K. G., Vaghela, P., & Ghosh, A. (2018). Differential growth, yield and biochemical responses of maize to the exogenous application of Kappaphycus alvarezii seaweed extract, at grain-filling stage under normal and drought conditions. *Algal Research*, *35*, 236–244. https://doi.org/10.1016/j.algal.2018.08.027
179. Ullah Asad, H., Wasim Haider, M., Muhammad Ayyub, C., Aslam Pervez, M., Manan, A., Ali Raza, S., & Ashraf, I. (2012). Impact of foliar application of seaweed extract on growth, yield and quality of potato (Solanum tuberosum L.). *Soil Environ*, *31*(2), 157–162. www.se.org.pkhttp://www.sss-pakistan.org
180. Vaghela, P., Trivedi, K., Anand, K. G. V., Brahmbhatt, H., Nayak, J., Khandhediya, K., Prasad, K., Moradiya, K., Kubavat, D., Konwar, L. J., Veeragurunathan, V., Grace, P. G., & Ghosh, A. (2023). Scientific basis for the use of minimally processed homogenates of Kappaphycus alvarezii (red) and Sargassum wightii (brown) seaweeds as crop biostimulants. *Algal Research*, *70*. https://doi.org/10.1016/j.algal.2023.102969
181. Velasco-Ramirez, A., Velasco-Ramírez, A. P., Hernández-Herrera, R. M., Garcia-Contreras, F. M., & Maldonado-Villegas, M. M. (2020). Effect of liquid seaweed extract on potted growth of Eustoma grandiflorum (Raf.) Shinners). In *Tropical and Subtropical Agroecosystems* (Vol. 23).
182. Vignesh, M., Ravindran, C., Kumar, S., Kalpana, K., Priya, L. S., Sundharaiya, K., Nageswari, K., Preethi, T. L., Manivannan, R., & Muthuramaligam, S. (2024). Optimizing growth conditions and biostimulant application for enhanced growth, yield and quality in Butter bean (Phaseolus lunatus L.) cultivar KKL 1. *Plant Science Today*, *11*(4), 74–82. https://doi.org/10.14719/pst.4516
183. Villa e Vila, V., Marques, P. A. A., Rezende, R., Wenneck, G. S., Terassi, D. de S., Andrean, A. F. B. A., Nocchi, R. C. de F., & Matumoto-Pintro, P. T. (2023). Deficit Irrigation with Ascophyllum nodosum Extract Application as a Strategy to Increase Tomato Yield and Quality. *Agronomy*, *13*(7). https://doi.org/10.3390/agronomy13071853
184. Villa e Vila, V., Piedade, S. M. D. S., Bouix, C. P., Rezende, R., Wenneck, G. S., Terassi, D. de S., Matumoto-Pintro, P. T., & Marques, P. A. A. (2024). Use of a Biostimulant Based on Seaweed Extract as a Sustainable Input to Enhance the Quality of Solanaceous Seedlings. *Horticulturae*, *10*(6). https://doi.org/10.3390/horticulturae10060642
185. Villa e Vila, V., Rezende, R., Marques, P. A. A., Wenneck, G. S., Nocchi, R. C. de F., Terassi, D. de S., Andrean, A. F. B. A., & Matumoto-Pintro, P. T. (2023). Seaweed extract of Ascophyllum nodosum applied in tomato crop as a biostimulant for improving growth, yield and soil fertility in subtropical condition. *Journal of Applied Phycology*, *35*(5), 2531–2541. https://doi.org/10.1007/s10811-023-03060-6
186. Vojnović, Đ., Maksimović, I., Tepić Horecki, A., Milić, A., Šumić, Z., Žunić, D., Adamović, B., & Ilin, Ž. (2024). Biostimulants Improve Bulb Yield, Concomitantly Affecting the Total Phenolics, Flavonoids, and Antioxidant Capacity of Onion (Allium cepa). *Horticulturae*, *10*(4). https://doi.org/10.3390/horticulturae10040391
187. Wadas, W., & Dziugieł, T. (2019). Growth and marketable potato (Solanum tuberosum l.) tuber yield in response to foliar application of seaweed extract and humic acids. *Applied Ecology and Environmental Research*, *17*(6), 13219–13230. https://doi.org/10.15666/aeer/1706_1321913230
188. Wadas, W., & Dziugieł, T. (2020a). Changes in assimilation area and chlorophyll content of very early potato (Solanum tuberosum L.) cultivars as influenced by biostimulants. *Agronomy*, *10*(3). https://doi.org/10.3390/agronomy10030387
189. Wadas, W., & Dziugieł, T. (2020b). Quality of new potatoes (Solanum tuberosum L.) in response to plant biostimulants application. *Agriculture (Switzerland)*, *10*(7), 1–13. https://doi.org/10.3390/agriculture10070265
190. Wang, Z., Yang, R., Liang, Y., Zhang, S., Zhang, Z., Sun, C., Li, J., Qi, Z., & Yang, Q. (2022). Comparing Efficacy of Different Biostimulants for Hydroponically Grown Lettuce (Lactuca sativa L.). *Agronomy*, *12*(4). https://doi.org/10.3390/agronomy12040786
191. Wasim Haider, M., Ullah Asad, H., Manan, A., & Ali Raza, S. (2012). *Impact of foliar application of seaweed extract on growth, yield and quality of potato (Solanum tuberosum L.)*. http://www.sss-pakistan.org
192. Wolski, K., Biernacik, M., Świerszcz, S., Talar-Krasa, M., & Leshchenko, O. (2019). Effect of the application of a biostimulant and mineral fertilizers on the concentration of mineral elements in the sward of forage mixtures cultivated on light soil. *Journal of Elementology*, *24*(1), 385–397. https://doi.org/10.5601/jelem.2018.23.2.1569
193. Yogendra, N. D., Prakhyath, K. M., Padalia, R. C., & Ghosh, A. (2024). Application of seaweed liquid extract improves the growth, yield, and chemical constituents of lemongrass. *Journal of Plant Nutrition*, *47*(19), 3571–3584. https://doi.org/10.1080/01904167.2024.2380777
194. Zamljen, T., Šircelj, H., Veberič, R., Hudina, M., & Slatnar, A. (2024). Impact of Two Brown Seaweed (Ascophyllum nodosum L.) Biostimulants on the Quantity and Quality of Yield in Cucumber (Cucumis sativus L.). *Foods*, *13*(3). https://doi.org/10.3390/foods13030401
195. Zapata-García, S., Temnani, A., Berríos, P., Espinosa, P. J., Monllor, C., & Pérez-Pastor, A. (2024). Optimizing Crop Water Productivity in Greenhouse Pepper. *Agronomy*, *14*(5). https://doi.org/10.3390/agronomy14050902
196. Zarzecka, K., Gugała, M., Sikorska, A., Grzywacz, K., & Niewęgłowski, M. (2020). Marketable yield of potato and its quantitative parameters after application of herbicides and biostimulants. *Agriculture (Switzerland)*, *10*(2). https://doi.org/10.3390/agriculture10020049
197. Zhang, Q., Masabni, J., & Niu, G. (2024). Microbial Biostimulants and Seaweed Extract Synergistically Influence Seedling Growth and Morphology of Three Onion Cultivars. *Horticulturae*, *10*(8). https://doi.org/10.3390/horticulturae10080800
198. Zodape, S. T., Kawarkhe, V. J., Patolia, J. S., & Warade, A. D. (n.d.). *Effect of liquid seaweed fertilizer on yield and quality of okra 1115 Effect of liquid seaweed fertilizer on yield and quality of okra (Abelmoschus esculentus L.)*.
